# DC‐magnetometry Analytical Tool Driven by Spin Ordering Phenomena for Sensing Chemical Interactions at the Surface of Nanomaterials

**DOI:** 10.1002/advs.202524213

**Published:** 2026-02-27

**Authors:** Marco Sanna Angotzi, Valentina Mameli, Alessandra Fantasia, Cesare Atzori, Giovanni Bertoni, Vincenzo Grillo, Giorgio Divitini, Dominika Zakutna, Jana Vejpravova, Martin Kalbac, Jan Plšek, Carla Cannas

**Affiliations:** ^1^ Department of Chemical and Geological Sciences University of Cagliari Cittadella Universitaria Monserrato Italy; ^2^ Consorzio Interuniversitario Nazionale per la Scienza e Tecnologia dei Materiali (INSTM) Cagliari Unit Firenze Italy; ^3^ European Synchrotron Radiation Facility Grenoble France; ^4^ CNR – Istituto Nanoscienze Modena Italy; ^5^ Istituto Italiano Di Tecnologia Genova Italy; ^6^ Department of Inorganic Chemistry Faculty of Science Charles University Prague Czech Republic; ^7^ Institut Laue‐Langevin – The European Neutron Source Grenoble France; ^8^ Department of Condensed Matter Physics Faculty of Mathematics and Physics Charles University Prague Czech Republic; ^9^ J. Heyrovský Institute of Physical Chemistry Czech Academy of Sciences Prague Czech Republic

**Keywords:** akaganeite, arsenic, interface science, magnetism, surface spin

## Abstract

DC‐magnetometry is proposed as an ultrasensitive probe to directly study surface spin ordering induced by chemical interactions. As a proof‐of‐concept, we investigated the adsorption of arsenic species onto akaganeite nanorods, a nanostructured iron oxyhydroxide particularly effective in the removal of As^V^/As^III^ species. DC‐magnetometry unequivocally revealed the presence of arsenate through a new and distinct magnetic signature (a second peak in the ZFC curve), highlighting the effect of the adsorbate‐adsorbent interaction. This peculiar magnetic feature, strictly dependent on the adsorbate speciation (As^III^ vs As^V^), amount, and type of interaction, was found to be directly and linearly correlated with the amount of As^V^ adsorbed on the surface. The comparison between the results obtained by different conventional and advanced techniques further highlighted the unique ability of DC magnetometry in qualitatively and quantitatively revealing the adsorption phenomenon. The suggestion of a ligand exchange process by XPS for the As^V^ allowed us to interpret the observed surface spin ordering in terms of specific chemical interactions. This evidence establishes DC‐magnetometry as a powerful and unconventional analytical tool for monitoring and quantifying complex surface phenomena on magnetically responsive materials suitable as heterogeneous catalysts, sorbents, and sensors.

## Graphical abstract

The adsorption of As^V^ on akaganeite nanorods was found to provide a magnetic signature. A change in the surface spin order gave rise to a second peak in the zero‐field cooled curve. As^III^ adsorption does not provide any change in the magnetic behavior of the sorbent. DC‐magnetometry can be used as direct quantitative probe of adsorbate‐adsorbent interactions.

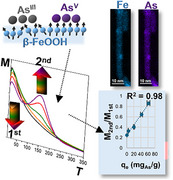

## 1 Introduction

Iron‐based materials, especially iron oxides and oxyhydroxides, have been used by humans since prehistory as pigments and then as precursors of iron artifacts, due to their natural abundance, chemical, and physical properties. Sixteen compounds are known among the generally called iron oxides, i.e., those involving Fe bond to O and/or OH groups. Different polymorphs with formula FeOOH do exist: goethite (α‐FeOOH), akaganeite (β‐FeOOH), lepidocrocite (γ‐FeOOH), feroxyhyte (δ’‐FeOOH), δ‐FeOOH, and high‐pressure FeOOH. In addition, poorly crystalline phases such as schwertmannite (Fe_16_O_16_(OH)_y_(SO_4_)_z_ · *n*H_2_O) and ferrihydrite (Fe_5_HO_8_ · 4H_2_O) deserve to be mentioned. The intriguing properties of iron oxyhydroxides, such as minute particles/crystallites, low degree of crystalline order, high eco‐ and biocompatibility, variety of magnetic properties, and surface area often higher than 100 m^2^ g^−1^ made them fascinating and effective nanostructured materials for many applications from biomedicine to catalysis and environmental remediation [1]. They are easily synthesized via scalable precipitation methods from soluble Fe salts, as a further advantage. In recent decades, the dramatic rise in global concern over water safety and environmental sustainability and the pressing need to ensure access to clean water has intensified global research on advanced materials for pollutant remediation. Thus, these abundant, low‐cost, and environmentally compatible iron‐based materials have become one of the most attractive of the transition metals. Thanks to the high surface area, surface hydroxyl groups, and charge, iron oxyhydroxides emerged as robust arsenic adsorbents [2]. Their adsorption ability is governed by inner‐sphere complexation through ligand exchange in the coordination spheres of surface structural iron atoms of the surface hydroxyl groups, with the formation of monodentate mononuclear or bidentate binuclear complexes, depending on the specific iron oxide [3, 4].

In this context, akaganeite exhibits exceptional arsenic uptake capacities and stability across a wide pH range [2, 5, 6] with a bidentate binuclear complex formation [4]. Among the iron oxides, akaganeite has peculiar structural properties: the anions are arranged in a body‐centered cubic cell, instead of a hexagonal or cubic close packing, resembling the hollandite‐type crystal structure. The Fe^III^ ions are octahedrally coordinated, with octahedra forming double chains through edge‐sharing and running parallel to the *b* axis. Each unit cell contains a central tunnel formed by corner sharing between the double chains and stabilized by chloride (or fluoride) anions, thus leading to the general formula (FeO(OH)_1−x_(Cl_x_), where x = 2–7 mol% [1].

As previously mentioned, iron oxides exhibit a wide range of magnetic behaviors: from antiferromagnetism (goethite, akaganeite, hematite) to speromagnetism (ferryhydrite) and ferrimagnetism (feroxyhyte, magnetite, maghemite) [1]. Actually, the magnetic behavior of each phase can be even more complex with transition from one magnetic regime to another depending on the experimental conditions, such as temperature, surface spin phenomena, or structural defects. Akaganeite is generally described as paramagnetic (or superparamagnetic by some authors [7, 8, 9] depending on the particles’ shape and synthesis method and due to the frustration of the antiferromagnetic order) at room temperature. It becomes antiferromagnetic below the Néel temperature (T_N_) of 240–300 K [9, 10, 11, 12, 13, 14], due to the presence of two spin sublattices antiferromagnetically coupled parallel to the 1D tunnels (along the b direction of the monoclinic I2/m structure) [9]. The value of T_N_ and the strength of the magnetic interactions are variable and depend upon synthesis conditions, i.e., the temperature and the length of the hydrolysis period, and associated with changes in the morphological properties of the particles (size and shape) and on the amount of interstitial water/chlorine anions in the compound. Curiously, some authors described the shift of T_N_ down to 10–20 K [15]. Weak ferromagnetism and exchange bias phenomena were described by different authors [10, 14], whereas others suggested a speromagnetic behavior [16, 17].

In the framework of adsorption studies, a proper characterization of the physical and chemical features of the sorbent is mandatory to fully understand its performance and to study the adsorption processes by investigating the adsorbent‐adsorbate interactions. Many techniques have been applied with this scope: XRD, UV–vis, FTIR, Raman, XPS, EDXS, DLS/ELS, SEM/FESEM, TEM, AFM, physisorption and porosimetry, thermoanalytical analysis (TG, DTA, DSC, and DTG). To the best of our knowledge, magnetometry has been used so far to characterize the magnetic properties of the pristine sorbent with the aim of exploit them for magnetic separation [18], whereas ^57^Fe Mössbauer Spectroscopy was rarely adopted to compare the properties of the iron‐bearing sorbent before and after its use [19].

Recently, we compared the arsenic uptake of different iron oxides and oxyhydroxides with akaganeite acting as the best sorbent in the whole pH range for arsenate anions and an effective one for arsenious acid [6]. In this work, we focused on this sorbent to compare the properties of the pristine and “after use” states. In particular, besides the application of characterization techniques with different sensitivity, resolution and depth sensing ability, such as powder X‐ray diffraction (PXRD), high‐resolution (scanning) transmission electron microscopy (HRSTEM), analytical electron microscopy (EDXS, EELS), X‐ray photoelectron spectroscopy (XPS), X‐ray absorption (XAS), and ^57^Fe Mössbauer Spectroscopy, DC magnetometry, was applied as a direct technique to study the adsorption uptake through possible changes in the magnetic response deriving from the adsorbate‐adsorbent interactions.

## 2 Results and Discussion

The adsorption of arsenic species on the akaganeite nanorods (Table S1) was found to occur without relevant changes in the chemical, structural, morphological, and textural properties of the sorbent (please refer to the Supporting Information for the complete characterization of the materials by powder X‐ray diffraction, N_2_ physisorption, X‐ray absorption spectroscopy, ^57^Fe Mössbauer spectroscopy, and transmission electron microscopy: Figures S1–S15 and Tables S2–S6). Besides a substantial homogeneous distribution of arsenic all over the sorbents upon adsorption, no additional crystalline phases (e.g., ferric arsenate, FeAsO_4_) or changes in the chemical coordination or oxidation state of iron ions were revealed and, in agreement with no changes in their surface area, the shape and size of the nanorods were also substantially kept.

**FIGURE 1:**
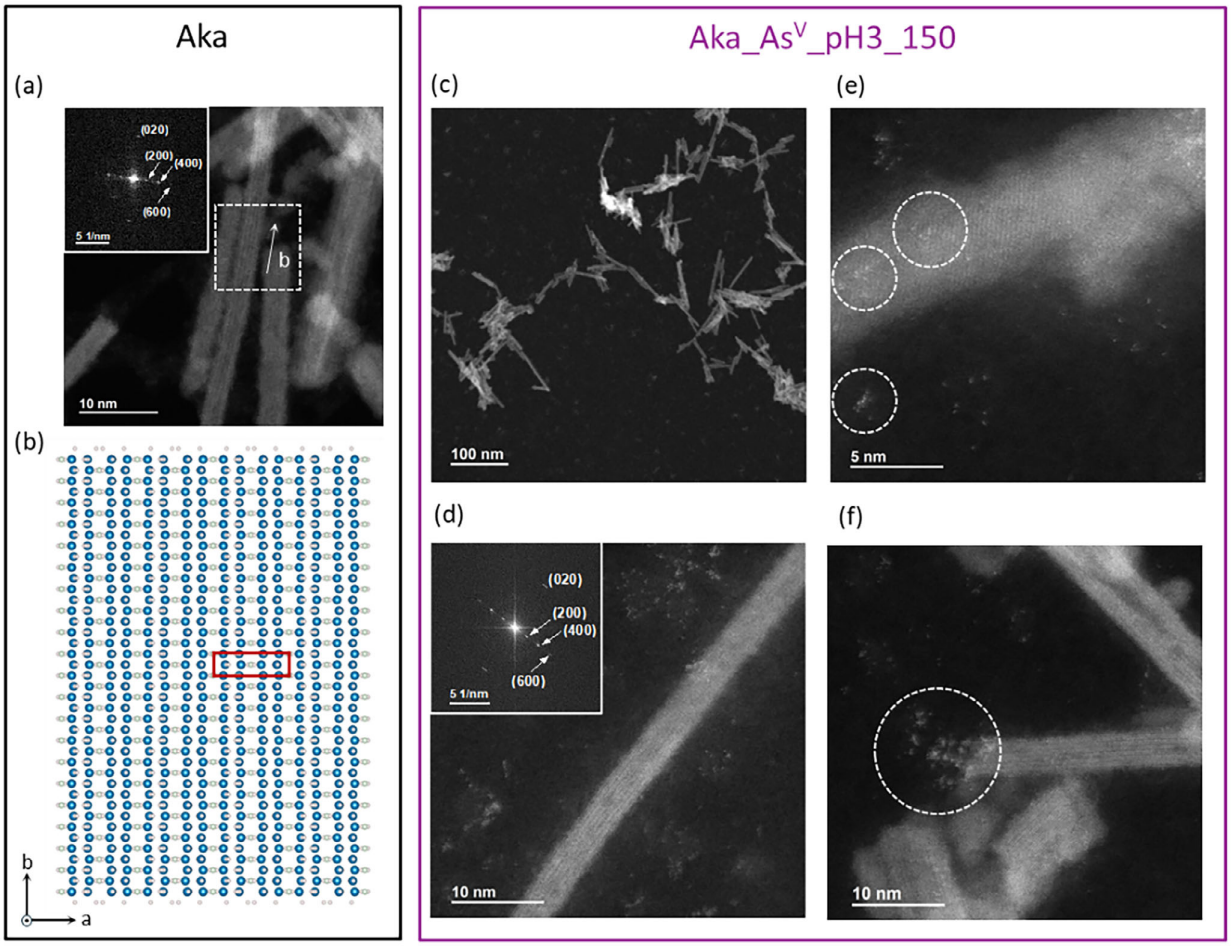
HAADF‐STEM images for the akaganeite before and after treating an As^V^‐spiked aqueous solution (C_As_ ≈ 150 mg L^−1^, pH ≈ 3): Aka (black frame) and Aka_As^V^_pH3_150 (purple frame), respectively. a) The nanorods grow along the *b* direction, as revealed by the diffractogram in the inset. b) Sketch of the akaganeite structure of the nanorods. In ADF‐STEM, the contrast is dominated by the heavier Fe atoms (blue) in the structure. c) Low magnification image showing the morphology of the nanorods after As treatment. d) The structure of the nanorods is well preserved. e,f) Bright spots corresponding to As clusters or adatoms from the solution are visible in the images also on the support carbon film of the TEM grid.

**FIGURE 2:**
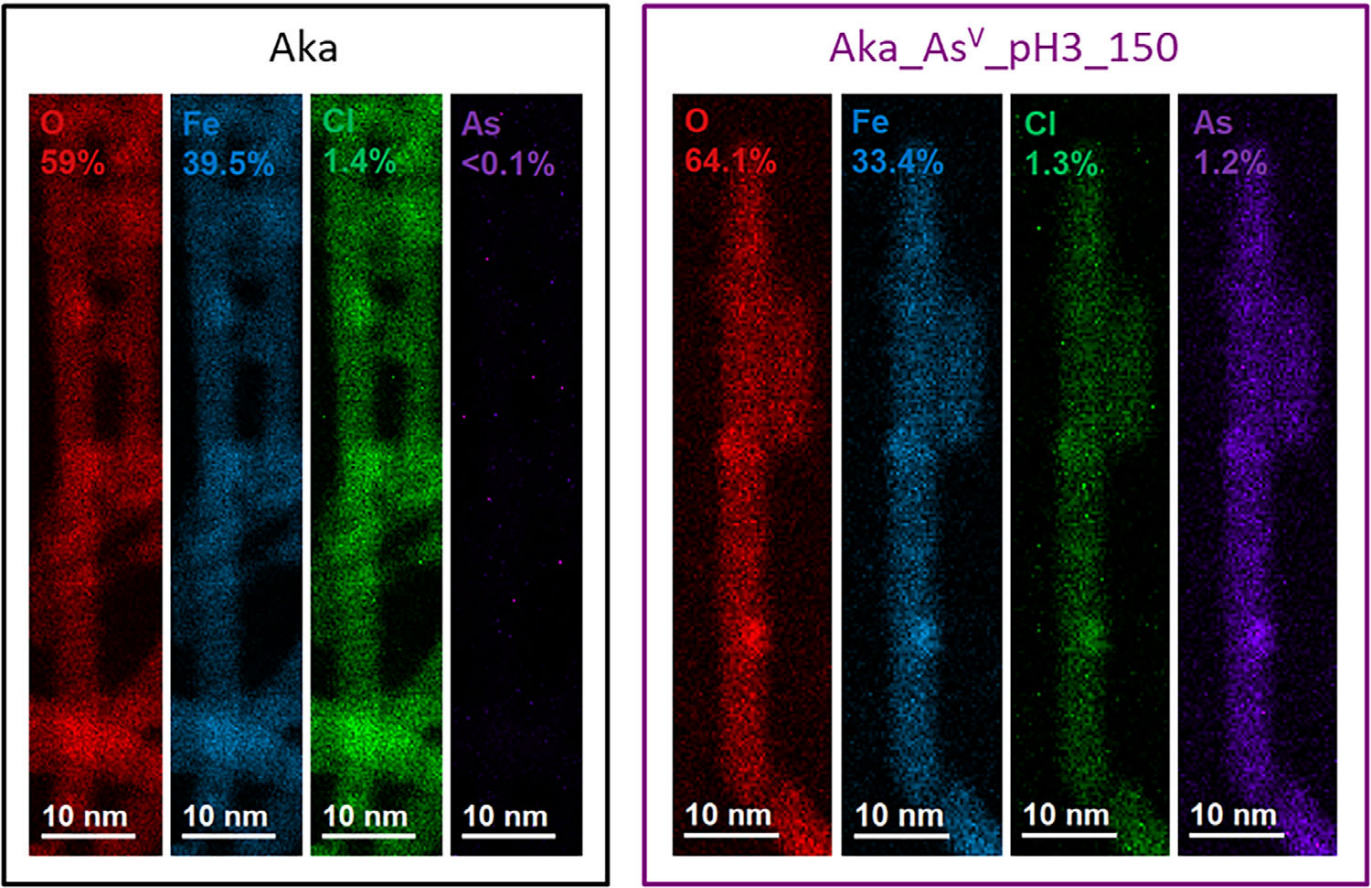
STEM‐EDXS chemical maps for the akaganeite before and after treating As^V^‐spiked aqueous solutions (C_As_ ≈ 150 mg L^−1^, pH ≈ 3): Aka (black frame) and Aka_As^V^_pH3_150 (purple frame), respectively. The amount in % are referred to these individual maps, while in the text we report the average values obtained from larger regions containing several nanorods.

Therefore, to investigate the chemical composition and in particular, the arsenic distribution at high magnification, HAADF‐STEM was coupled with EDXS and EELS in STEM mode. The analysis was conducted on the 150 mg L^−1^ As^V^ sample, which was the one with the highest adsorbed arsenic amount and low free arsenic in solution (As C_e_ values reported in Table S1). ADF‐STEM images (Figure 1) reveal the presence of highly crystalline wires of akaganeite growing in the *b* directions, together with bright spots that could be excess of adatoms of arsenic surrounding the wires (white circles) or the spherical particles or can be related to free arsenic in solution. The low concentration of arsenic signal made detection and quantification particularly challenging, especially in EELS. The As‐L signal at ∼1330 eV is hidden below the background produced by the Fe‐L signal (Figure S14), and while it was visible on average EEL spectra using very long acquisition time, extracting a distribution map was not possible. For this reason, EDXS was performed by taking advantage of the higher rate of X‐ray emission due to the as atomic weight (Figure S15), and the acquired spectra were further denoised using principal component analysis [20], by keeping the components with significant variance. The resulting elemental map demonstrates As is homogeneously distributed along the nanowires (Figure 2).

The chemical composition on the Aka_As^V^_pH3_150 sample reveals a Fe/O atomic ratio of 0.55 extracted from EELS, a Cl/Fe of 0.04 from EDXS, consistent with the expected formula for akaganeite (FeO(OH,Cl)). The Cl/Fe value is lower with respect to the value obtained for Aka (0.16). This difference might be ascribed to the contact of the sample with the As‐spiked aqueous solution during the 16‐h removal test; thus part of chloride ions was probably washed away. The As/Fe atomic ratio was measured from EDXS and EELS, resulting in 0.045(3) and 0.039(7), respectively.

In this framework, since no relevant changes were detected as a consequence of the adsorbate‐adsorbent interactions, the samples were further investigated by DC magnetometry by different protocol measurements, being expected to be potentially sensitive even to phenomena occurring at the surface or involving a small fraction of iron ions.

**FIGURE 3:**
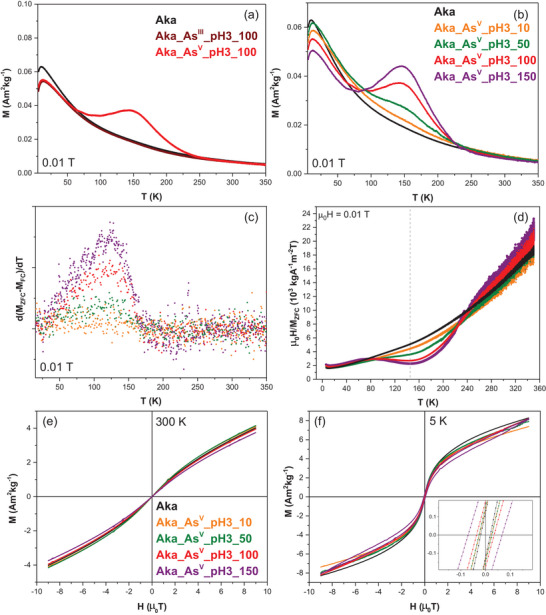
ZFC curves (a,b), T_B_ distributions (c), inversed field‐normalized ZFC magnetization as a function of the temperature (d), field‐dependent magnetization curves at 300 (e) and 5 K (f) of the akaganeite before and after treating As^III^‐ and As^V^‐spiked aqueous solutions with different As concentrations.

First, the magnetic behavior of the pristine akaganeite was checked through ZFC‐FC protocols at 0.01 T (Figure 3a reporting only ZFC curves, Figure S16a reporting both ZFC and FC curves at two different magnetic field, namely 0.01 and 0.0025 T): the ZFC and FC curves appeared overlapped until a temperature value of about 150 K, when a bifurcation was observed together with a small peak at about 10 K in the ZFC curve (Table 1). This behavior was explained in the literature for akaganeite nanowires of 50 nm in length as typical of an antiferromagnet with the occurrence of spin‐freezing phenomena due to structural disorder and other effects related to the presence of canted spin [15]. The authors observed that the peak in the ZFC curve shifted from 13 K for the 50 nm nanowires to 20 K for all the other‐sized nanowires (120, 160, and 200 nm). The magnetic isotherms at 5, 150, and 300 K showed the progressive loss of the small hysteresis loop associated with the presence of canted spins toward a paramagnetic trend (i.e., a positive linear increase of the magnetization as a function of the applied magnetic field with the loss of coercivity, Figure S16b), as expected based on the ZFC curve, and in agreement with the previously mentioned study on the 50 nm‐sized nanowires [15]. Contrarily, other authors observed the presence of an hysteresis loop both at 150 and 300 K, ascribed by the authors to the presence of a disordered surface spin structure [10].

**TABLE 1:** Magnetic parameters of the akaganeite before and after treating As^V^‐spiked aqueous solutions with different As concentrations. The data for the reference sample Aka_AsV_6% are also given for comparison.

Sample	H_c_ (5 K) (mT)	M_9T_ (5 K) (Am^2^kg^−1^)	M_7T_ (5 K) (Am^2^kg^−1^)	M_9T_ (300 K) (Am^2^kg^−1^)	M_7T_ (300 K) (Am^2^kg^−1^)	T_max_ (K)	T_B_ (K)
Aka	19(1)	8.3(4)	7.5(4)	4.0(2)	3.4(2)	10.5(5)	—
Aka_As^V^_pH3_10	24(1)	7.4(4)	6.7(3)	4.1(2)	3.5(2)	13.6(7) 150(30)	—
Aka_As^V^_pH3_50	26(1)	8.1(4)	7.2(4)	4.2(2)	3.5(2)	13.5(7) 150(20)	—
Aka_As^V^_pH3_100	36(2)	8.2(4)	7.2(4)	3.9(2)	3.3(2)	12.9(6) 140(7)	120(6)
Aka_As^V^_pH3_150	68(3)	8.2(4)	7.2(4)	3.7(2)	3.1(2)	12.9(6) 145(7)	120(6)
Aka_As^V^_6%	47(2)	—	7.6(4)	—	3.6(2)	27(1)	—

Upon As^III^ adsorption, no relevant changes were revealed, except for a lowering of the magnetization of the FC curve, which got closer to the ZFC one, and a shift of the peak at about 13.7 K (Figure 3a for the ZFC curves, Figure S17 for both ZFC and FC curves). Surprisingly, As^V^ anions induced a previously unobserved magnetic feature, consisting in the appearance of a second peak in the ZFC curve at about 145 K, besides the shift toward 13–14 K of the first peak associated with the spin‐freezing phenomena (Figure 3a and Figure S17) [15]. This behavior was further confirmed by recording the ZFC‐FC curves for the sample Aka_As^V^_pH8_100 (Figure S28) and by applying a more intense external magnetic field (0.1 T), but no shift in the position of the peaks was detected (Figure S18). To verify if this feature was related to the arsenate adsorption, other samples recovered after the treatment of the As^V^‐spiked solution with different initial As concentration were analyzed under the same experimental conditions: the second peak was present in all the ZFC curves with an associated magnetization value dependent on the expected arsenic amount at the surface of the sorbent (Figure 3b for the ZFC curves, Figures S19 and S20 for both ZFC and FC curves). Moreover, based on the absence of a change in the magnetic response upon As^III^ adsorption contrarily to what observed for As^V^, the findings suggested a weak As^III^‐akaganeite interaction, probably due to the presence of neutral arsenious acid molecules, and indicated that the adopted approach is sensitive to the speciation and to the oxidation processes As^III^→As^V^, that were proved to do not occur during our experiments. From the first derivative of the difference between the magnetization values of the ZFC and FC curves for the entire temperature profile, the critical temperature was estimated to be around 120 K for the Aka‐As^V^_pH3_100 and Aka‐As^V^_pH3_150 samples (Figure 3c and Table 1). In addition, the magnetization was also studied as a function of the applied magnetic field by magnetic isotherms at 5 and 300 K for all samples (Figure 3e,f). If, on the one hand, in the 300 K the curves are almost completely overlapped and no further information can be extracted from them, on the other hand, the presence of a small hysteresis loop in the 5 K isotherms revealed the presence of non‐zero magnetic moments due to canted spins and the related coercivity was found to increase with increasing adsorbed arsenic amount. Moreover, a progressive opening of the hysteresis loop was found in the Aka_As^V^_pH3_100 and Aka_As^V^_pH3_150 samples.

The possible existence of linear dependence of the magnetic parameters from the amount of As adsorbed per unit mass of solid sorbent (q_e_) was also verified. A good linear trend (R^2^ = 0.9873) was achieved for the ratio of the magnetization of the second peak with respect to the first peak in the ZFC curve (Figure S21).

The observed behavior seems to be justified by the ordering process of the canted spins at the surface of the akaganeite nanorods and not by bulk phenomena. To confirm this hypothesis, a reference sample (Aka_As^V^_6%) of akaganeite synthesized in the presence of arsenate anions (as described in the experimental section) to achieve a mass percentage of about 6%, close to that of the Aka_As^V^_pH3_100 sample, was fully characterized (Figures S22–S25). This sample resembles the crystal structure of akaganeite, but it is expected to incorporate arsenic species not just at the surface of the sample but in the bulk. Indeed, since arsenate was present during the coprecipitation synthesis process, its incorporation is expected to occur during nucleation and crystal growth, leading to a bulk‐distributed iron‐arsenic species rather than a preferentially surface‐localized one [21]. The ^57^Fe Mössbauer spectrum of this sample, the related hyperfine parameters, and the magnetic behavior were different from that of the Aka_As^V^_pH3_100 sample. No second peak was present in the ZFC curve, and the first peak appeared at about 27 K suggesting that the reason behind the second peak is the bond of the arsenate species with the surface iron cations in the exhausted sorbent. The magnetic characterization of the Aka_As^V^_pH3_250 sample (Figure S26) revealed no further increase in the magnetization of the second band with respect to the first one in the ZFC curve, suggesting that the increase in the adsorption uptake does not involve a direct bond to the surface iron ions, thus suggesting that DC magnetometry may distinguish between different layers of adsorbed species.

**FIGURE 4:**
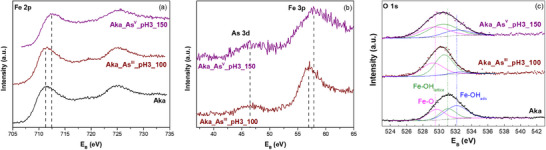
XPS spectra of Fe 2p (a), As 3d and Fe 3p (b), O 1s (c) for the akaganeite before and after the treatment of As^III^‐ and As^V^‐spiked aqueous solutions (C_As_ ≈ 100–150 mg L^−1^, pH ≈ 3).

Since the observed behavior was ascribed to surface phenomena due to the adsorbate‐adsorbent interactions, X‐ray photoelectron spectroscopy (Figure 4) was applied to the samples with the highest and similar (100–150 mg L^−1^) adsorbed arsenic amount and low free arsenic in solution for the two oxidation states (As C_e_ values reported in Table S1). This technique was able to detect changes in the chemical environment of iron ions, but only when the As^V^ species were adsorbed at the surface of akaganeite similarly to DC magnetometry, revealing a shift in the Fe 2p bands (from 711.4 eV, a typical value for Fe^3+^ in FeOOH polymorphs [22, 23], to 712.6 eV for the Fe 2p3/2, Figure 4a and Table S7). This shift was confirmed by a similar shift observed for the Fe 3p band (at about 57 eV) for the sample Aka_As^V^_pH3_150 with respect to the Aka_As^III^_pH3_100 (Figure 4b and Table S7). The broad As 3d band (at about 46 eV, as expected from the literature [24]) featured a low intensity due to the low amount of arsenic and no shift was observed despite the different oxidation state (Figure 4b and Table S7). Unfortunately, the broadening and low intensity of the As 3d band hampered the possibility to distinguish the oxidation states or to get further information on the features of the bonds, as previously reported by other authors [24, 25].

Other authors observed a similar shift in the Fe 2p band position but also a change in the profile of the O 1s band upon As adsorption, which was described by adding different contributions both for the pristine and the As^V^‐adsorbed sample [22, 26]. In particular, Zhang et al. [26] described the O 1s band for the akaganeite before the adsorption as made up of four bands located around 529.7, 531.0, 531.9, and 533.4 eV. The first two bands were assigned to the lattice oxygen atoms binding with Fe (Fe–O) and the lattice hydroxyl (Fe–OH_lattice_), whereas the other two bands were ascribed to adsorbed hydroxyl (Fe–OH_ads_) and water (H_2_O), respectively. After the adsorption of the As^V^ species, the band associated with the surface hydroxyl groups (531.9 eV) was found to decrease due to the ligand exchange process between the surface hydroxyl groups and As^V^ ions. Moreover, the first two bands (i.e., Fe–O, Fe–OH_lattice_) were found to reduce their intensity because of the adsorption process. Deliyanni et al. [22] reported a curve fitting of the O 1s band at 530.3 eV in an akaganeite sample modified by the use of hexadecyltrimethylammonium bromide by only three sub‐spectra. Two of them were centered at about 530.3 and 532.1 eV, which take into account the presence of the lattice oxygen of the oxide and the lattice oxygen of the hydroxyl groups. The comparison between these studies suggests that this latter contribution accounts for both lattice and adsorbed ‐OH groups. Moreover, a third contribution at 528.8 eV was reported for the oxygen atoms after the adsorption of the surfactant molecules. When the As^III^ species were adsorbed at the surface, the Fe 2p band was found to shift from 711 to 712.4 eV (similarly to our findings), whereas the O 1s band shifts to 531.4 eV and became broader. The authors suggested that the observed behavior can be explained by fitting the band with two subspectra instead of the original three accounting for the oxygen of the oxide (529.9 eV) and oxygen of the hydroxyl groups (532.2 eV).

Based on these previous data, in the present study, the O 1s band (Figure 4c and Table S8) was fitted by three subspectra assigned as lattice oxygen (Fe–O, at about 529 eV), lattice hydroxyl (Fe–OH_lattice_, at about 531 eV), and surface hydroxyl (Fe–OH_ads_, at about 532 eV). The fraction of Fe–OH (Fe–OH_lattice_ + Fe–OH_ads_) was 71% for pristine akaganeite, whereas after adsorption of As^III^/As^V^ species it decreased to 63% and 59% for Aka_As^III^_pH3_100 and Aka_As^V^_pH3_150 samples, respectively. In particular, the observed decrease was due to the significant decrease of the relative intensity of Fe–OH_ads_ due to the ligand exchange process, in agreement with the above‐mentioned studies [22, 26]. Since the binding energy of As–OH_lattice_ groups overlaps with Fe–OH_lattice_ groups [23], an increase of the component at 531 eV (see also the Fe–OH_lattice_/Fe–O ratio values reported in Table S8) can be partially caused by the presence of an additional unresolved component deriving from As–OH_lattice_ bonds.

To further investigate the microscopic nature of the surface spin ordering responsible for the second peak in the ZFC curve for the AsV‐loaded akaganeite samples, additional AC/DC magnetometry measurements by different protocols were performed (for all the details refer to the paragraphs entitled “DC/AC magnetometry” and “AC susceptibility analysis”, Figures S27–S36 and Tables S9 and S10). Among the different magnetic behavior reasonably associable to this magnetic signature, such as blocking process in superparamagnets (SPM), freezing process in spin glasses (SG), super spin glasses (SSG), SSG‐ and cluster glass (CG)‐like systems, the results seem to indicate a complex interacting CG like state, arising from the local disrupting of the main magnetic behavior due to the presence of As–O–Fe bonds at the surface forming small magnetic islands.

## 3 Conclusion

In this work, the study of the adsorbate‐adsorbent interactions was investigated by DC‐magnetometry, whose efficacy was demonstrated in akaganeite nanorods upon As^V^ and As^III^ adsorption. PXRD and XAS did not reveal the formation of additional phases than akaganeite or changes in the chemical environment of As and Fe species, while STEM‐EDXS confirmed the homogeneous distribution of As through the sorbent. ^57^Fe Mössbauer spectroscopy indicated a slight change in the electric field experienced by the iron ions, with changes in the quadrupole splitting. Most notably, DC magnetometry revealed, only in the case of As^V^, an additional peak in the ZFC curve for the As‐loaded samples with respect to the pristine akaganeite, whose corresponding magnetization correlated linearly with the amount of adsorbed arsenic and independently on the pH of the As removal test. Therefore, this magnetic behavior was ascribed to spin ordering phenomena due to the adsorbate‐adsorbent interaction occurring at the surface of the sorbent, interpreted as a consequence of the ligand exchange process between bonded –OH groups with arsenate ones, suggested by the XPS results through a change in the chemical environment of surface iron atoms. Concerning As^III^, no changes were detected upon adsorption by DC magnetometry, suggesting that the weaker adsorbate‐adsorbent interaction involving electrically neutral species (i.e., arsenious acid) does not induce any spin ordering phenomena. This was further confirmed by XPS, showing correspondingly no changes in the chemical environment of iron atoms.

These results highlight the unique sensitivity of magnetic measurements to surface interactions and suggest a new role for DC magnetometry as a direct analytical tool for probing adsorption processes and chemical reactions occurring at the surface of magnetically responsive materials, through a magnetic response that is not only dependent on the adsorbate amount, but also on the speciation and on the environmental conditions, such as the pH. This approach could be extended to other adsorbate–adsorbent systems, offering a powerful approach for environmental remediation and water treatment, but also for all those applications involving sorption phenomena, including gas purification, capture, and sensing. Nevertheless, although the highlighted promising perspective on the use of DC‐magnetometry, also thanks to the low amount of sample needed, it should be mentioned that further technological challenges to be faced with will be associated with the costs of the instrument equipment, time‐consuming measurements, due to the cooling process of the ZFC protocols.

## 4 Materials and Methods

### 4.1 Synthesis

The pristine akaganeite (**Aka**) was synthesized as described elsewhere [6], starting from a literature procedure but with some modification [27]. In a 100 mL borosilicate bottle with a polypropylene cap, 12.5 mL of 0.2 M EDTA was added to 28.5 mL of 5.26 M sodium hydroxide solution. To this solution, 25 mL of 2 M FeCl_3_·6H_2_O solution was added (pH 10) under vigorous stirring. The pH was adjusted to 2 with the addition of HCl 37% w/w, and the suspension was aged at 98°C for 4 h in a laboratory oven. The bottle was then rapidly cooled in an ice bath. The solid was separated through centrifugation at 7000 rpm, washed several times with water, and then with ethanol until the chloride content was considered as structural (Cl/Fe = 0.11, estimated by STEM‐ EDXS analysis) [28], then collected and dried under air at 55°C for 2 days.

After different aliquots of the **Aka** sample were used as sorbents to treat different As‐spiked aqueous solutions as described in our previous study [6], the solid samples were also recovered and dried at mild temperature (40°C) in an oven. These samples, referred to as “after use” samples, will be named as **Aka_As^x^_pHy_z**, where **x** identifies the arsenic species (V, III), y is the pH of the adsorption test (= 3 or 8), and z is the initial concentration of the As‐spiked solution.

In addition, an akaganeite sample was also synthesized in the presence of As^V^ aqueous species by mixing the two precursor solutions, i.e., FeCl_3_·6H_2_O and sodium arsenate. This sample was used as reference for the measurements of the chemical‐physical properties of the “after‐use” akaganeite samples, and it was synthesized to achieve a similar As/Fe ratio of the Aka_As^V^_pH3_100. Table S1 summarizes the samples under study.

### 4.2 Powder X‐ray diffraction (PXRD)

The samples were characterized by powder X‐ray diffraction (PXRD, Figure S1‐S2), using a PANalytical X'Pert PRO with Cu Kα radiation (1.5418 Å), a secondary monochromator, and a PIXcel1D position‐sensitive detector. The data were collected in the 20°–80° of 2θ with the step size of 0.05°. The diffraction position and instrumental width were calibrated using powder LaB_6_ from NIST. Some of the measurements were confirmed by repeating the analysis with another diffractometer (Seifert X3000 with a scintillation detector). Refinement of structural parameters was obtained by the Rietveld method, through MAUD software, adopting recommended fitting procedures.

### 4.3 N_2_ Physisorption

Textural analyses of all samples were performed on a Micromeritics ASAP 2020 (Micromeritics, Norcross, Georgia, USA) by determining the nitrogen adsorption‐desorption isotherms at −196°C. Prior to analyses, the samples were heated for 12 h under a vacuum at 60°C (heating rate, 1°C min^−1^). The specific surface area (S_BET_) was computed by the Brunauer–Emmett–Teller (BET) equation [29]. The total pore volume (V_p_) was calculated at p/p^0^ = 0.95. The pore diameter was determined by applying the Barrett–Joyner–Halenda (BJH) model [30] to the isotherm desorption branch, although both adsorption and desorption pore size distributions are reported in the Supporting Information together with the textural properties (Figure S3, Table S2).

### 4.4 X‐ray absorption spectroscopy (XAS)

Experimental X‐ray absorption spectroscopy (XAS) data at the As and the Fe K‐edge were acquired at the BM23 beamline of the ESRF [31] in transmission mode (Figure S4). Optimized amounts of powdered samples were pressed into 5 mm diameter pellets using a die and a hydraulic press. To enhance the signal‐to‐noise ratio, particularly in EXAFS mode, multiple spectra were acquired and averaged/summed for each sample, covering the energy range of the two K‐edges +500 eV. A Fe or an Au (due to its L‐III edge) foil were positioned after the sample served as a reference for calibrating the collected data. Data processing of the X‐ray absorption near‐edge structure (XANES) was carried out using Athena software, while data fitting was performed with the Artemis plugin, FEFF. Both software programs are included in the Demeter package developed by Ravel et al. [32].

### 4.5 ^57^Fe Mössbauer Spectroscopy

Room temperature ^57^Fe Mössbauer spectroscopy was performed on a Wissel spectrometer using transmission arrangement and proportional detector LND‐45431 (Figure S5). An α‐Fe foil was used as a standard, and the fitting procedure was done by the NORMOS program to determine the isomer shift (δ), quadrupole splitting (Q_s_), full width at half maximum (FWHM), depth (D), and relative integrated area (A) (Table S3, Figure S6). The fitting of hyperfine parameters was performed using a standard least‐squares fitting method with Lorentzian spectral lines.

### 4.6 Transmission electron microscopy (TEM) and STEM‐EDXS/EELS

The specimens for transmission electron microscopy (TEM) analysis were prepared by dropping an ethanol dispersion of the samples on a 200‐mesh carbon‐coated copper grid. TEM micrographs at low magnification were obtained using a JEOL JEM 1400 Plus operating at 100 kV (Figure S7, S11). Chemical mapping and line profiles were obtained at low magnification by energy dispersive X‐ray spectroscopy (EDXS) in scanning TEM (STEM) mode (Figure S8–S10, S12‐S13, Table S4‐S6). High‐resolution images were obtained in scanning mode using a high‐angle annular dark field detector (HAADF‐STEM) on a Thermo Fisher Scientific Spectra 300 microscope operated at 300 kV and equipped with a probe aberration corrector. The elemental maps on the nanorods were acquired using EDXS by means of a double detector to improve signal to noise ratio (Figure S15). Due to the very low amount of As with respect to Fe, a denoising of the spectra was performed with principal component analysis (PCA), to enhance the signals in the elemental maps. The presence of As in the treated sample was confirmed with electron energy‐loss spectroscopy (EELS) using a Gatan, Inc. energy filter (Figure S14).

### 4.7 DC Magnetometry

DC magnetic properties were studied on powders with a Quantum Design PPMS DynaCool (µ_0_H_max_ = 9 T) system by using the VSM module (Figure S16–S26). Different kinds of magnetic measurements were carried out. The field dependence of the magnetization (M vs H) was studied at 5, 150, and 300 K between 9 and −9 T. The temperature dependence of magnetization (M vs T) was studied by using the zero‐field‐cooling (ZFC) and field‐cooling (FC) protocols: the sample was cooled down to 6 K in a zero magnetic field; then, the signals were recorded under a static magnetic field. M_ZFC_ was measured during the warm‐up from 6 to 350 K, whereas M_FC_ was recorded during the cooling step. For the data reported in the paragraphs entitled “DC/AC magnetometry” and “AC susceptibility analysis” of the Supporting Information (Figure S27–S36, Table S9–S10), different magnetometers were adopted: a SQUID MPMS3XL and a PPMS9 equipped with a VSM, both from Quantum Design. All the details on the adopted protocols are described in those paragraphs.

### 4.8 X‐ray photoelectron spectroscopy (XPS)

The X‐ray photoelectron spectroscopy (XPS) measurements were performed in a VG ESCA3 MkII electron spectrometer with a base pressure better than 10^−9^ mbar (Table S7‐S8). The powder samples were mounted on a double‐sided adhesive tape. Al Kα radiation was used for the excitation of the electrons. The electrons were energy analyzed using a hemispherical analyzer operating at constant pass energy of 20 eV. The spectra were calibrated by setting C 1s peak belonging to adventitious carbon on the sample surface to the binding energy of 284.8 eV.

### 4.9 Statistical Analysis

No data transformation or normalization procedures were applied prior to analysis. Data for arsenic adsorption capacity and chemical composition were presented as mean values ± standard deviation, when applicable. For particle size analysis, average values were extracted from large regions containing several particles to ensure representativeness. Magnetic parameters were reported with associated experimental uncertainties derived from the instrument sensitivity and data fitting procedures. The linear dependence of magnetic parameters as a function of the amount of adsorbed arsenic were evaluated using ordinary least squares regression, with the goodness of fit expressed by the coefficient of determination (R^2^). For the determination of the analytical detection limit, a confidence factor of 3 was applied to the reagent blank signal, corresponding to a 98.3% confidence level. Data processing and statistical evaluations were performed using OriginPro and Microsoft Excel software.

## Conflicts of Interest

The authors declare no conflicts of interest.

## Supporting information




**Supporting File**: advs74570‐sup‐0001‐SuppMat.docx.

## Data Availability

The data that support the findings of this study are available from the corresponding author upon reasonable request.
