# Supplementary material for: DC‐magnetometry Analytical Tool Driven by Spin Ordering Phenomena for Sensing Chemical Interactions at the Surface of Nanomaterials
Source: Adv Sci (Weinh). 2026 Feb 27;13(30):e24213. doi: 10.1002/advs.202524213 (PMC13248821; doi:10.1002/advs.202524213)
Supplement: Supplementary file 1 — Supporting File: advs74570‐sup‐0001‐SuppMat.docx. [file ADVS-13-e24213-s001.docx]

Supporting Information for

**DC-magnetometry Analytical Tool Driven by Spin Ordering Phenomena for Sensing Chemical Interactions at the Surface of Nanomaterials**

Marco Sanna Angotzi,^a,b^ Valentina Mameli,^a,b^ Alessandra Fantasia,^a,b^ Cesare Atzori,^c^ Giovanni Bertoni,^d^ Vincenzo Grillo,^d^ Giorgio Divitini,^e^ Dominika Zakutna,^f^ Jana Vejpravova,^f,g^ Martin Kalbac,^h^ Jan Plšek,^h^ Carla Cannas^a,b*^

^a^Department of Chemical and Geological Sciences, University of Cagliari, Cittadella Universitaria, S.S. 554 bivio per Sestu, 09042, Monserrato, Italy

^b^Consorzio Interuniversitario Nazionale per la Scienza e Tecnologia dei Materiali (INSTM), Cagliari Unit, Via Giusti 9, 50121, Firenze, Italy

^c^European Synchrotron Radiation Facility, 71 Avenue des Martyrs, CS 40220, 38043 Cedex 9, Grenoble, France

^d^CNR - Istituto Nanoscienze, Via G. Campi 213/A, 41125, Modena, Italy
^e^Istituto Italiano di Tecnologia, Via Morego 30, 16163, Genova, Italy

^f^Department of Inorganic Chemistry, Faculty of Science, Charles University, Hlavova 2030/8, 128 43 Prague 2, Prague, Czech Republic

^g^Department of Condensed Matter Physics, Faculty of Mathematics and Physics, Charles University, Ke Karlovu 3, 121 16 Prague 2, Prague, Czech Republic

^h^J. Heyrovský Institute of Physical Chemistry, Czech Academy of Sciences, Dolejškova 2155/3, 182 23, Prague, Czech Republic

[Table S1 List of the akaganeite samples analysed in the manuscript. 5](#_Toc221296610)

[Powder X-ray Diffraction (PXRD) and nitrogen physisorption analysis 6](#_Toc221296611)

[Figure S1 PXRD patterns (a) and N_2_ physisorption isotherms (b) for the akaganeite before (Aka, black curve) and after treating As^III^- and As^V^-spiked aqueous solutions (C_As_ ≈ 100 mg L^-1^; pH ≈ 3). 6](#_Toc221296612)

[Figure S2 PXRD patterns for the akaganeite before (Aka, black curve) and after treating As^III^- (a) and As^V^-spiked (a, b) aqueous solutions (C_As_ ≈ 100 mg L^-1^, 150 mg L^-1^, 250 mg L^-1^, 500 mg L^-1^; pH ≈ 3). 7](#_Toc221296613)

[Figure S3 Pore size distribution obtained from the adsorption (a) and desorption (b) branches of the N_2_ physisorption isotherms of the akaganeite before (Aka, black curve) and after treating As^III^- and As^V^-spiked aqueous solutions (C_As_ ≈ 100 mg L^-1^; pH ≈ 3). 7](#_Toc221296614)

[Table S2 Textural properties of the akaganeite before (Aka, black curve) and after treating As^III^- and As^V^-spiked aqueous solutions (C_As_ ≈ 100 mg L^-1^; pH ≈ 3). 7](#_Toc221296615)

[X-ray absorption spectroscopy (XAS) 8](#_Toc221296616)

[Figure S4 EXAFS and XANES spectra at the K edge of Fe (a, b) and As (c, d) for the akaganeite before and after treating As^III^- and As^V^-spiked aqueous solutions (C_As_ ≈ 100 mg L^-1^, pH ≈ 3). 8](#_Toc221296617)

[^57^Fe Mӧssbauer spectroscopy 9](#_Toc221296618)

[Figure S5 ^57^Fe Mössbauer spectra of the akaganeite before and after treating As^III^- and As^V^-spiked aqueous solutions with different As concentrations. 10](#_Toc221296619)

[Table S3 Mössbauer hyperfine parameters obtained by curve fitting of the ^57^Fe Mössbauer spectra of the akaganeite before and after treating As^III^- and As^V^-spiked aqueous solutions with different As concentrations and at pH 3: isomer shift (δ), quadrupole splitting (Q_s_), full width at half maximum (FWHM), depth (D), relative integrated area (A). For comparison, the data for the reference sample Aka_As^V^_6% are also listed. 11](#_Toc221296620)

[Figure S6 Plots of quadrupole splitting values for the 1^st^ and 2^nd^ doublet as a function of the amount of As adsorbed per unit mass of solid sorbent (q_e_) determined by ICP-OES analysis of the treated solution. 12](#_Toc221296621)

[Transmission electron microscopy (TEM) 13](#_Toc221296622)

[Figure S7 Conventional TEM micrographs for the akaganeite before and after treating As^III^- and As^V^-spiked aqueous solutions (C_As_ ≈ 100 mg L^-1^, pH ≈ 3): Aka (black), Aka_As^III^_pH3_100 (wine red) and Aka_As^V^_pH3_100 (light red), respectively. 13](#_Toc221296623)

[Figure S8 EDXS chemical mapping of the Aka_As^III^_pH3_100 sample at low magnification. 14](#_Toc221296624)

[Figure S9 EDXS linear profile of the Aka_As^III^_pH3_100 (a) and Aka_As^V^_pH3_100 (b) samples at low magnification. 14](#_Toc221296625)

[Figure S10 EDXS chemical mapping of the Aka_As^V^_pH3_100 sample at low magnification. 14](#_Toc221296626)

[Table S4 EDXS data for the chemical mapping and linear profile of the Aka_As^III^_pH3_100 sample. 15](#_Toc221296627)

[Table S5 EDXS data for the chemical mapping and linear profile of the Aka_As^V^_pH3_100 sample. 15](#_Toc221296628)

[Figure S11 Conventional TEM micrographs for the akaganeite after treating As^V^-spiked aqueous solutions (C_As_ ≈ 500 mg L^-1^, pH ≈ 3). 16](#_Toc221296629)

[Figure S12 EDXS chemical mapping of the Aka_As^V^_pH3_500 sample at low magnification. 16](#_Toc221296630)

[Figure S13 EDXS linear profiles of the Aka_As^V^_pH3_500 sample at low magnification. 16](#_Toc221296631)

[Table S6 EDXS data for the chemical mapping and linear profile of the Aka_As^V^_pH3_500 sample. 16](#_Toc221296632)

[High-angle annular dark field detector (HAADF-STEM) in scanning TEM (STEM) mode 17](#_Toc221296633)

[Figure S14 Average EEL spectrum from the Aka_AsV_pH3_150 sample (acquisition time: 100 s). The O-K, Fe-L and As-L edge are quantified with Hartree-Slater models and a power law background. Intensity is in log-scale to better show the As signal at ∼1330 eV. Quantification results were obtained by averaging 3 different regions in the sample. 17](#_Toc221296634)

[Figure S15 Average EDXS spectrum from one nanorod of the Aka_AsV_pH3_150 sample. The As/Fe ratio resulted 0.045(3) in good agreement with EELS data. 17](#_Toc221296635)

[DC magnetometry 18](#_Toc221296636)

[Figure S16 Zero field cooled – field cooled (ZFC-FC) curves recorded under 0.0025 and 0.01 T of external magnetic field (a) and magnetic isotherms at 5 K, 150 K, and 300 K (b) for the pristine akaganeite sample. 18](#_Toc221296637)

[Figure S17 ZFC-FC curves of the pristine akaganeite and of the Aka_As^V^_pH3_100 and Aka_As^III^_pH3_100 samples. 18](#_Toc221296638)

[Figure S18 ZFC-FC curves normalised (left) and absolute magnetisation values (right) for the Aka_As^V^_pH3_100 akaganeite sample recorded under different applied magnetic fields (0.01 T, 0.1 T). 18](#_Toc221296639)

[Figure S19 ZFC-FC curves of the pristine akaganeite (Aka, black lines) and of the akaganeite samples recovered after treating As^III^- and As^V^-spiked aqueous solutions with different As concentrations. 19](#_Toc221296640)

[Figure S20 ZFC curves (left) and ZFC-FC curves (right) of the pristine akaganeite (Aka, black lines) and of the akaganeite samples recovered before and after treating As^III^- and As^V^-spiked aqueous solutions with different As concentrations presented as separate curves. 20](#_Toc221296641)

[Figure S21 Linear dependence of different magnetic parameters from the amount of As adsorbed per unit mass of solid sorbent (q_e_). 21](#_Toc221296642)

[Characterization of the reference sample Aka_As^V^_6% 22](#_Toc221296643)

[Figure S22 Powder XRD pattern (a) and Rietveld analysis (b) of the reference sample Aka_As^V^_6%. 22](#_Toc221296644)

[Figure S23 Bright field (a-d) and dark field (e) TEM micrographs and selected area electron diffraction (f) of the reference sample Aka_As^V^_6%. 22](#_Toc221296645)

[Figure S24 ^57^Fe Mössbauer spectrum of the reference sample Aka_As^V^_6%. 23](#_Toc221296646)

[Figure S25 Zero field cooled – field cooled curves (a) and magnetic isotherms at 300 K (b) and 5 K (c) of the reference sample Aka_As^V^_6%. 24](#_Toc221296647)

[Magnetic characterization of the sample Aka_As^V^_pH3_250. 25](#_Toc221296648)

[Figure S26 Magnetic characterization of the Aka_As^V^_pH3_250 sample. 25](#_Toc221296649)

[X-ray photoelectron spectroscopy (XPS) 26](#_Toc221296650)

[Table S7 XPS data extracted from the XPS spectra of the akaganeite before and after the treatment of As^III^- and As^V^-spiked aqueous solutions (C_As_ ≈ 100, 150 mg L^-1^, pH ≈ 3). N.A. stands for “*not acquired*”. 26](#_Toc221296651)

[Table S8 XPS data acquired by fitting the O 1s band of the XPS spectra of the akaganeite before and after the treatment of As^III^- and As^V^-spiked aqueous solutions (C_As_ ≈ 100, 150 mg L^-1^, pH ≈ 3). 26](#_Toc221296652)

[DC/AC magnetometry 27](#_Toc221296653)

[Figure S27 ZFC-FC curves of the sample Aka_H_2_OmilliQ_pH3 at a magnetic field of 0.01 T. 27](#_Toc221296654)

[Figure S28 ZFC-FC curves for the Aka_As^V^_pH8_100 sample in comparison with those of the Aka_As^V^_pH3_100 sample at two different magnetic field values (0.01 T, 0.1 T). 27](#_Toc221296655)

[Figure S29 ZFC-FC curves recorded under different magnetic field (0.01 T - 0.5 T) on the sample Aka_As^V^_pH3_100 by a SQUID magnetometer (MPMS3XL from Quantum Design). 28](#_Toc221296656)

[Figure S30 ZFC-FC curves recorded under different magnetic field (0.01 T - 0.5 T) on the sample Aka_As^V^_pH3_100 by a SQUID magnetometer (MPMS3XL from Quantum Design). 29](#_Toc221296657)

[Figure S31 In-phase (χ') and out-of-phase (χ'') AC susceptibility as a function of temperature (5-240 K) at different frequencies (1-999 Hz) on the sample Aka_As^V^_pH3_100 recorded by a SQUID magnetometer (MPMS3XL from Quantum Design). 29](#_Toc221296658)

[Figure S32 Memory effect experiments recorded on the sample Aka_AsV_pH3_100 by a VSM of a PPMS (PPMS9 from Quantum Design). 30](#_Toc221296659)

[Figure S33 Thermoremanent magnetization (TRM) curve with 1-hour stops at 200 K and 100 K during the cooling process recorded on the sample Aka_AsV_pH3_100 by a VSM of a PPMS (PPMS9 from Quantum Design). 30](#_Toc221296660)

[Figure S34 Isothermal remanent magnetization (IRM) curves at different temperatures (5 K, 100 K, 300 K) recorded on the sample Aka_AsV_pH3_100 by a SQUID magnetometer (MPMS3XL from Quantum Design). 30](#_Toc221296661)

[AC susceptibility analysis 32](#_Toc221296662)

[Table S9 Temperature of the maxima of the in-phase (χ') AC susceptibility for the given attempt frequency. 32](#_Toc221296663)

[Figure S35 Fit of the data extracted from the maxima of the in-phase (χ') AC susceptibility by the Arrhenius and Vogel-Fulcher laws. 33](#_Toc221296664)

[Figure S36 Fit of the data extracted from the maxima of the in-phase (χ') AC susceptibility by the Arrhenius and Vogel-Fulcher laws with the limits for the expected range of relaxation times (upper bound for τ = 10^-9^ s). 33](#_Toc221296665)

[Table S10 Equations used for the AC susceptibility analysis. 34](#_Toc221296666)

[References 35](#_Toc221296667)

Table S1 List of the akaganeite samples analysed in the manuscript.

| **Sample** | **Description** | **As C_0_ (mg/kg)** | **As C_e_ (mg/kg)** | **As_Ads_ (wt%)** | **q_e_ (mg/g)** | **As/Fe^1^ (wt%)** | **As/Fe^2^ (wt%)** |
| --- | --- | --- | --- | --- | --- | --- | --- |
| **Aka** | *Pristine* akaganeite sample | *N.A.* | *N.A.* | *N.A.* | *N.A.* | *N.A.* | *N.A.* |
| **Aka_As^V^_6%** | Akaganeite sample precipitated in the presence of AsV aqueous species (*reference sample*) | *N.A.* | *N.A.* | *N.A.* | *N.A.* | *0.064* | *N.A.* |
| **Aka_As^V^_pH3_10** | Akaganeite samples recovered after their use to treat arsenic spiked aqueous solutions  (*“after use” samples*) | 10 | 0 | 99.7 | 4.3 |  | 0.007 |
| **Aka_As^V^_pH3_50** |  | 46.8 | 0 | 99.9 | 20.2 |  | 0.032 |
| **Aka_As^V^_pH3_100** |  | 95.5 | 0.2 | 99.9 | 41.8 | 0.056 | 0.067 |
| **Aka_As^V^_pH3_150** |  | 145.3 | 3.9 | 97.3 | 61.7 |  | 0.098 |
| **Aka_As^V^_pH3_250** |  | 255.6 | 162.9 | 36.3 | 77.5 |  | 0.123 |
| **Aka_As^V^_pH3_500** |  | 469.9 | 281.5 | 40.1 | 79.8 |  | 0.127 |
| **Aka_As^III^_pH3_100** |  | 103.9 | 19.5 | 81.8 | 35.8 | 0.065 | 0.057 |
| **Aka_As^III^_pH3_500** |  | 567.1 | 355.8 | 37.3 | 90.7 |  | 0.144 |
| **Aka_As^V^_pH8_100** |  | 98.4 | 0.8 | 99.2 | 42.3 |  | 0.067 |
| ^1^ Determined by ICP-OES on the digested solid sample.  ^2^ Determined by ICP-OES as difference between the initial concentration of the untreated solution and the equilibrium concentration of the solution recovered after the adsorption batch test. | | | | | | | |

# Powder X-ray Diffraction (PXRD) and nitrogen physisorption analysis

**Figure S1a** reports the PXRD patterns of the pristine akaganeite, hereafter labelled Aka, in comparison with the samples recovered after the treatment of As^III^- and As^V^-spiked solutions with an initial concentration of arsenic around 100 mg L^-1^ and at pH 3, named Aka_As^III^_pH3_100 (q_e_ = 35.8 mg_As_/g_sorbent_, **Table S1**) and Aka_As^V^_pH3_100 (q_e_ = 41.8 mg_As_/g_sorbent_): no relevant changes were revealed as a consequence of the adsorption of arsenic species at the surface of akaganeite, with only diffraction peaks ascribable to the monoclinic I2/m space group (PDF card #00-042-1315). Contrarily, other authors reported the presence of additional bands at 22° and 54° due to the precipitation of ferric arsenate (FeAsO_4_).^[1]^ In order to further verify the formation of additional phases, the PXRD patterns (**Figure S2**) were also recorded for akaganeite samples used to treat more concentrated As-spiked solutions (*i.e.*, for As^V^: C_As_ = 150 mg L^-1^ (q_e_ = 61.7 mg_As_/g_sorbent_), 250 mg L^-1^ (q_e_ = 77.5 mg_As_/g_sorbent_), 500 mg L^-1^ (q_e_ = 79.8 mg_As_/g_sorbent_); for As^III^: 500 mg L^-1^ (q_e_ = 90.7 mg_As_/g_sorbent_): also in the case of these samples, only akaganeite was present.

The textural properties of Aka, Aka_As^III^_pH3_100, and Aka_As^V^_pH3_100 were studied by recording N_2_ physisorption isotherms (**Figure S1b, Table S2**). Similar isotherms of type IVa with a H2a hysteresis loop (according to IUPAC classification dated to 2015),^[2]^ similar values of surface areas (≈180-190 m^2^ g^-1^) and pore volumes (≈0.13 cm^3^ g^-1^) were observed for the samples. Notably, a mean pore size of about 3-4 nm was estimated for the samples by BJH model applied on the desorption branch for all samples. Nevertheless, the model applied on both the adsorption and desorption branches provides interesting feature (**Figure S3**): although the pore size distribution obtained from the desorption branches does not reveal relevant changes unless lower dV/dw values for the As-loaded samples, in the case of the adsorption branches upon As adsorption the distribution around 4 nm almost completely disappeared.

**
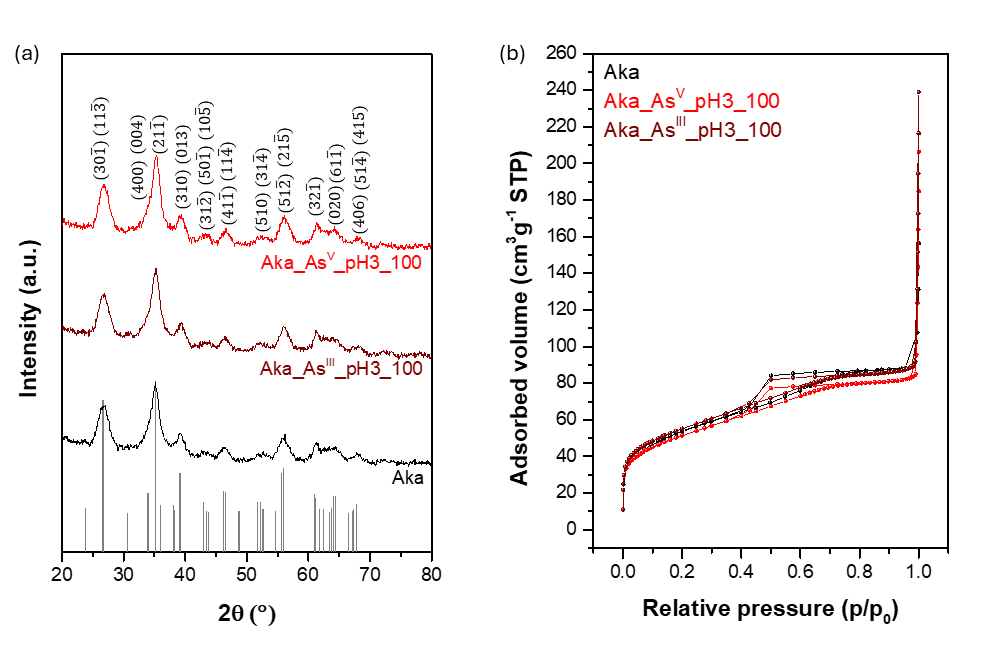
**

Figure S1 PXRD patterns (a) and N_2_ physisorption isotherms (b) for the akaganeite before (Aka, black curve) and after treating As^III^- and As^V^-spiked aqueous solutions (C_As_ ≈ 100 mg L^-1^; pH ≈ 3).


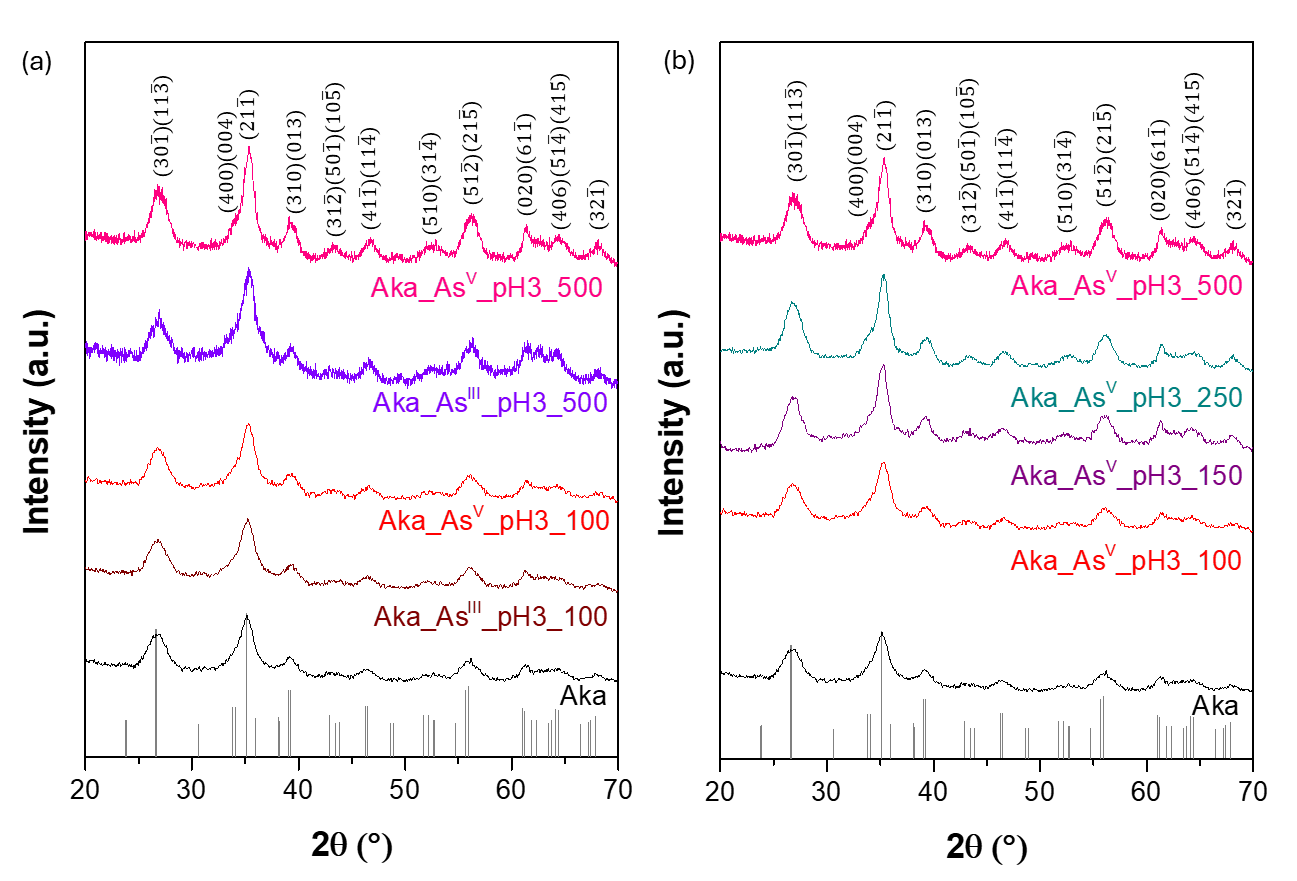


Figure S2 PXRD patterns for the akaganeite before (Aka, black curve) and after treating As^III^- (a) and As^V^-spiked (a, b) aqueous solutions (C_As_ ≈ 100 mg L^-1^, 150 mg L^-1^, 250 mg L^-1^, 500 mg L^-1^; pH ≈ 3).


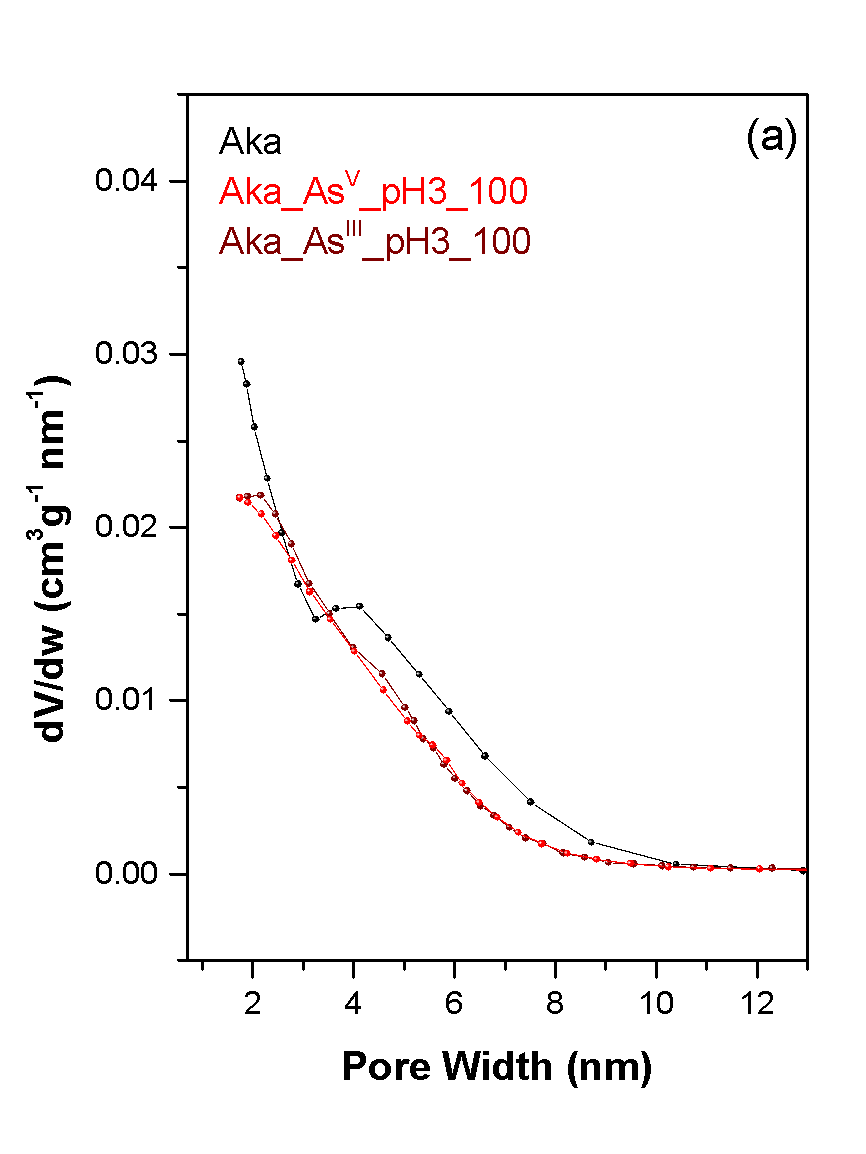

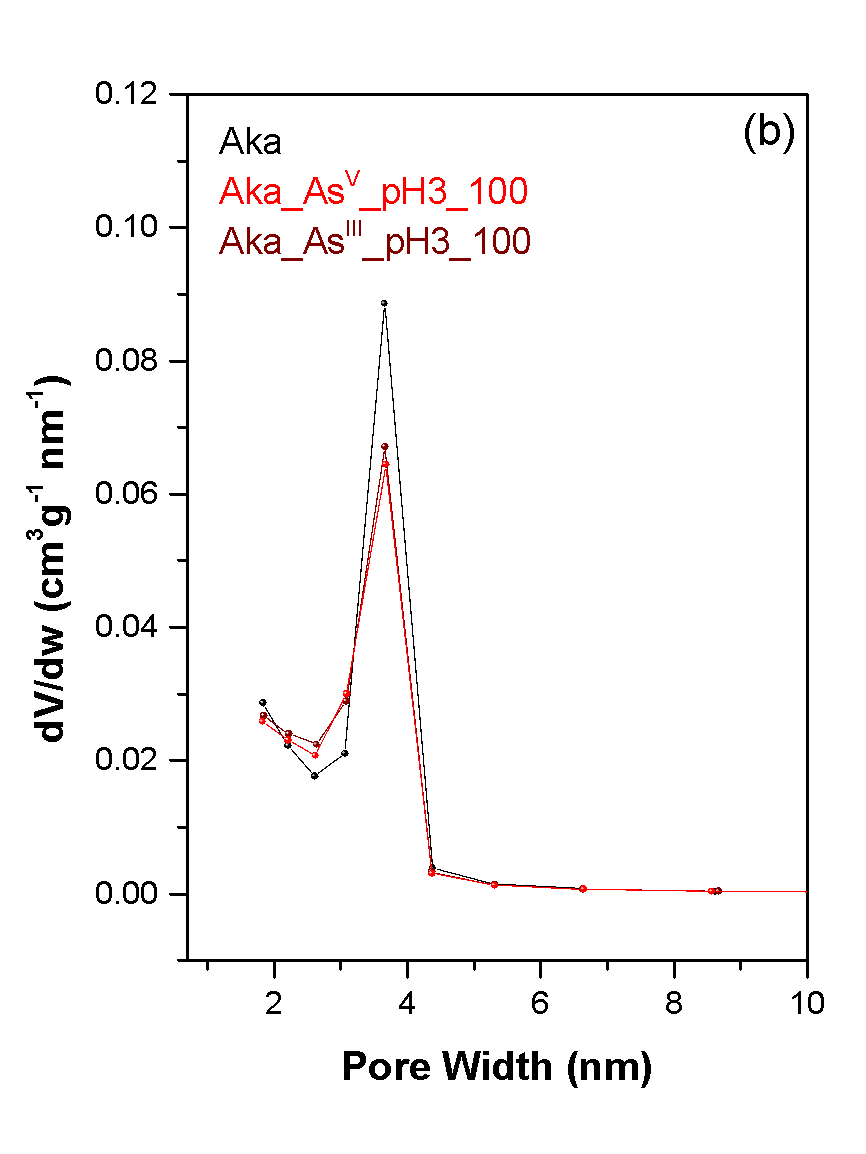


Figure S3 Pore size distribution obtained from the adsorption (a) and desorption (b) branches of the N_2_ physisorption isotherms of the akaganeite before (Aka, black curve) and after treating As^III^- and As^V^-spiked aqueous solutions (C_As_ ≈ 100 mg L^-1^; pH ≈ 3).

Table S2 Textural properties of the akaganeite before (Aka, black curve) and after treating As^III^- and As^V^-spiked aqueous solutions (C_As_ ≈ 100 mg L^-1^; pH ≈ 3).

| **Sample** | **S_BET_ (m^2^ g^-1^)** | **V_P_ (cm^3^ g^-1^)^*^** | **D_P_ (nm)^#^** |
| --- | --- | --- | --- |
| Aka | 185(4) | 0.136(1) | 3.17(7) |
| Aka_As^III^_pH3_100 | 190(4) | 0.135(1) | 3.91(8) |
| Aka_As^V^_pH3_100 | 180(4) | 0.127(1) | 3.81(8) |
| ^*^measured as single point. | | | |
| ^#^measured by BJH model on the desorption branch. | | | |

# X-ray absorption spectroscopy (XAS)

The acquired X-ray absorption spectra at the Fe K edge (**Figure S4a,b**) were found to almost perfectly overlap in the three samples, *i.e.* Aka, Aka_As^III^_pH3_100, and Aka_As^V^_pH3_100, both in the near edge and in the fine structure zones of the spectra. The Fe K-edge XANES is located at about 7133 eV and is ascribed to the 1s🡪4p electron transition, indicating the presence of Fe(III), with a pre-edge at 7115 eV, corresponding to a 1s🡪3d electron transition.^[3,4]^

The nearly perfect overlap of the Fe K-edge XANES and EXAFS spectra among pristine and arsenic-loaded samples indicates that the local coordination environment of Fe remains unaltered. This strongly supports the hypothesis that arsenic incorporation occurs predominantly at the surface, without affecting the bulk structure of akaganeite. Moreover, it contributes to exclude, in agreement with XRD data, the formation of additional phases, such as iron arsenate. On the contrary, as expected, the spectra at the As K edge (**Figure S4c**) revealed differences in the position of the edge and in the local environment of As atoms, with a noticeable shift in the absorption edge energy (11874.4 eV for Aka_As^V^_pH3_100 and 11870.8 eV for Aka_As^III^_pH3_100), indicating the difference in the oxidation state of As.^[5,6]^ The results suggest that the adsorption process resulted in the incorporation of As^V^ or As^III^ species within the surface akaganeite structure, mainly keeping their original oxidation state. The EXAFS analysis (**Figure S4d**) further corroborates the distinct local environments surrounding As in the two samples. Significant differences are observed in the EXAFS oscillations and their Fourier transforms, indicating variations in the types, distances, and/or coordination numbers of neighbouring atoms around the As species. These structural discrepancies clearly demonstrate that As occupies different sites, or experiences different bonding configurations, within the akaganeite lattice, depending on the prevailing oxidation state and/or incorporation mechanism. This is consistent with the XANES results.


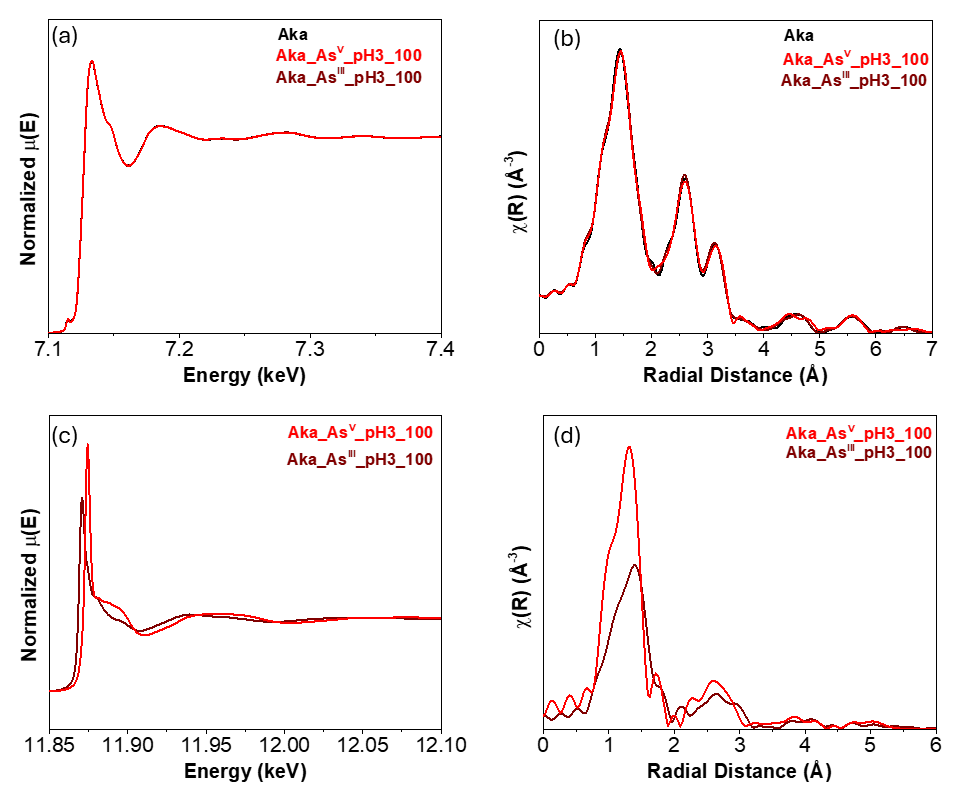


Figure S4 EXAFS and XANES spectra at the K edge of Fe (a, b) and As (c, d) for the akaganeite before and after treating As^III^- and As^V^-spiked aqueous solutions (C_As_ ≈ 100 mg L^-1^, pH ≈ 3).

# ^57^Fe Mӧssbauer spectroscopy

In this complex scenario, the presence of ^57^Fe isotopes in the material and its magnetic properties can be exploited. Therefore, ^57^Fe Mӧssbauer spectroscopy and DC magnetometry were applied to get further insights, as they previously allowed us to study even diluted iron oxide-bearing nanocomposites, both natural and synthetic.^[7,8]^ The room temperature (RT) ^57^Fe Mӧssbauer spectra are shown in **Figure S5** and the hyperfine parameters are listed in **Table S3**. The spectrum of the pristine akaganeite in its paramagnetic state was reported in a previous study,^[9]^ and it was fitted by two sub-spectra accounting for Fe^3+^ ions in two different sub-environments, as suggested in the literature.^[10]^ According to the literature, the reason behind the presence of two subspectra in the RT Mӧssbauer spectrum might be ascribed to two non-equivalent iron states, arising from distorted structure, ^[10,11]^ different coordination,^[12]^ or the replacement of Cl ions by OH ions.^[13]^

The obtained hyperfine parameters for the pristine akaganeite sample (Aka) well agreed with previously published values.^[10,11,14]^ Starting from this curve fitting, the spectra of the As-adsorbed samples were described with two doublets, whose hyperfine parameters were found to change slightly according to the As speciation and the As amount. In particular, an isomer shift of about 0.37-0.38 mm s^-1^ was obtained for all samples. Interestingly, a general decrease in the width of the doublets was observed in the samples after the As removal from 0.33 and 0.35 mm s^-1^ to 0.30 and 0.32 mm s^-1^ (mean values for the first and second doublet in the spectra of the As-samples, regardless the speciation and As amount), suggesting some ordering process due to the bonds with the As species. Concerning the quadrupole splitting, the values slightly decreased in the case of As^V^ anions with respect to the pristine akaganeite, whereas the As^III^ adsorption caused the opposite trend with a slight increase. Furthermore, plotting the quadrupole splitting values as a function of the amount of As adsorbed per unit mass of solid sorbent (q_e_) revealed an almost linear trend in the range 0 - 41.8 mg_As_ g_sorbent_^-1^, as shown in **Figure S6** for As^V^ removal. These findings, although the linear trend is not certain based on the errors associated with both the hyperfine parameters and arsenic uptake, suggest that slight but detectable change in the charge distribution around the iron ions occurs because of the adsorption process causing change in the electric field gradient that is connected to the size of quadrupole splitting. Villacorta *et al*.^[14]^ also studied the effect of arsenic anions adsorbed at the surface of akaganeite on the RT Mӧssbauer spectrum, for an arsenic removal batch test carried out under the following conditions: As^III^, C_0_ = 40 mg L^-1^, pH 2. Differently from our results, they did not observe any relevant change in the hyperfine parameters of the pristine and As^III^-loaded pure akaganeite sample, although slight changes, in terms of relative areas of the subspectra, were revealed in some cases when co-precipitated akaganeites (in the presence of 60 ppm of Hg, As and Sb) were used as adsorbents. The authors also discussed these changes in their study suggesting the involvement of three different factors: (i) surrounding atomic environment of the iron ions; (ii) the relative abundance of iron ions at the surface of akaganeites; and (iii) the types and distribution of active sites for adsorption. In particular, they explained the observed changes in the hyperfine parameters as the consequence of a change in the Fe 3d orbital population and ascribed the fact that these changes were just slight to the low relative abundance of iron ions at the surface and to the exposure of different active adsorption sites. Therefore, in our case, the observed effect in the changes of quadrupole splitting may suggest detectable changes in the Fe 3d orbital population and a favourable exposure of adsorption active sites.

Figure S5 ^57^Fe Mössbauer spectra of the akaganeite before and after treating As^III^- and As^V^-spiked aqueous solutions with different As concentrations.

Table S3 Mössbauer hyperfine parameters obtained by curve fitting of the ^57^Fe Mössbauer spectra of the akaganeite before and after treating As^III^- and As^V^-spiked aqueous solutions with different As concentrations and at pH 3: isomer shift (δ), quadrupole splitting (Q_s_), full width at half maximum (FWHM), depth (D), relative integrated area (A). For comparison, the data for the reference sample Aka_As^V^_6% are also listed.

| **Sample** | **Subspectrum** | **δ (mm/s)** | **Q_S_ (mm/s)** | **FWHM (mm/s)** | **D** | **A (%)** |
| --- | --- | --- | --- | --- | --- | --- |
| Aka | Doublet 1 | 0.373(1) | 0.536(7) | 0.330(7) | 0.095(4) | 57 |
|  | Doublet 2 | 0.379(2) | 0.940(9) | 0.346(8) | 0.071(4) | 43 |
| Aka_As^III^_pH3_100 | Doublet 1 | 0.371(2) | 0.554(4) | 0.318(5) | 0.054(1) | 64 |
|  | Doublet 2 | 0.3777(2) | 0.951(4) | 0.307(5) | 0.031(1) | 36 |
| Aka_As^V^_pH3_10 | Doublet 1 | 0.370(2) | 0.533(4) | 0.315(3) | 0.0218(2) | 61 |
|  | Doublet 2 | 0.376(2) | 0.941(6) | 0.314(5) | 0.0142(2) | 39 |
| Aka_As^V^_pH3_50 | Doublet 1 | 0.3705(6) | 0.526(1) | 0.290(1) | 0.0762(2) | 59 |
|  | Doublet 2 | 0.3747(9) | 0.930(2) | 0.312(3) | 0.0529(4) | 41 |
| Aka_As^V^_pH3_100 | Doublet 1 | 0.372(2) | 0.513(7) | 0.291(7) | 0.044(3) | 53 |
|  | Doublet 2 | 0.377(3) | 0.90(2) | 0.35(1) | 0.039(3) | 47 |
| Aka_As^V^_pH3_150 | Doublet 1 | 0.374(4) | 0.522(7) | 0.304(9) | 0.0110(4) | 58 |
|  | Doublet 2 | 0.385(6) | 0.90(1) | 0.32(1) | 0.0079(5) | 42 |
| Aka_As^V^_6% | Doublet 1 | 0.369(2) | 0.53(1) | 0.32(2) | 0.775(7) | 50 |
|  | Doublet 2 | 0.379(3) | 0.93(2) | 0.39(1) | 0.781(1) | 50 |

Figure S6 Plots of quadrupole splitting values for the 1^st^ and 2^nd^ doublet as a function of the amount of As adsorbed per unit mass of solid sorbent (q_e_) determined by ICP-OES analysis of the treated solution.

# Transmission electron microscopy (TEM)

By conventional TEM (**Figure S7**), the presence of akaganeite in the form of a predominant population of randomly oriented nanorods and a second population of very small spheroidal nanoparticles, as previously described,^[9]^ sometimes forming agglomerates, was found for all samples. The adsorption of arsenic did not result in changes in the morphology or size ratios of the akaganeite nanowires or increase in the extent of agglomeration phenomena. The akaganeite nanowires appear to be well-crystalline as highlighted by dark field imaging mode (**Figure S7b**). The EDXS chemical analysis was also performed through chemical mapping and line profiles on both Aka_As^III^_pH3_100 (**Figure S8**, **Figure S9**, **Table S4**), and Aka_As^V^_pH3_100 (**Figure S9**, **Figure S10**, and **Table S5**) at low magnification in scanning TEM mode (STEM). Arsenic appears homogeneously distributed in the samples (**Figure S8**, **Figure S10**) with a strong overlap of arsenic and iron in the line profile (**Figure S9**). If slight inhomogeneities are visible, they might be ascribed to the fact that not all the akaganeite surface is covered by the adsorbed arsenic species, taking into account that the initial arsenic concentration of about 100 mg L^-1^ is not sufficient to saturate the sorbent.^[9]^ These findings are further confirmed by the semi-quantitative data extracted from the maps and line profile (**Table S4**, **Table S5**). As/Fe values of about 0.05 and 0.085 are obtained as the ratio between the weight percentages for the Aka_As^III^_pH3_100 and Aka_As^V^_pH3_100, in agreement with those obtained by ICP-OES (0.06 and 0.07, respectively). To verify the role of the arsenic loading in obtaining information on the morphology and the distribution of arsenic over akaganeite, the Aka_As^V^_pH3_500 sample was also analysed by conventional TEM (**Figure S11**) and STEM-EDXS (**Figure S12**, **Figure S13**, **Table S6**). The TEM micrographs show similar features for this sample in comparison with those previously analysed, with the presence of slightly agglomerated nanorods. Homogeneous distribution of arsenic in the chemical map and completely overlapped line profiles were observed in this case, as expected due to the saturation of the sorbent at that initial arsenic concentration.^[9]^ Moreover, the estimated As/Fe ratio from the weight percentages was found to be about 0.18, slightly higher than the mean value estimated from ICP-OES (0.13), with this difference ascribable to the limited accuracy of standardless quantification.


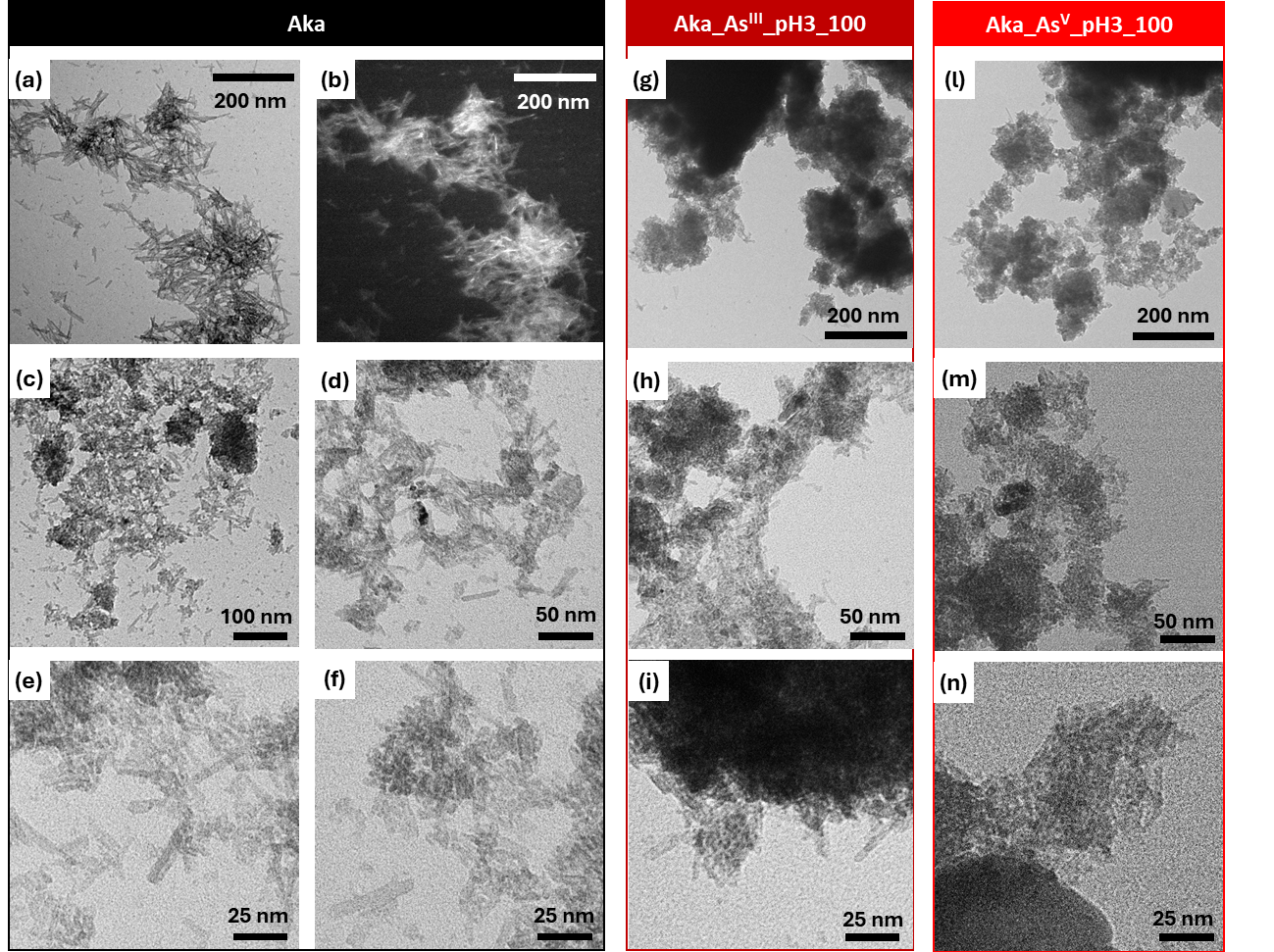


Figure S7 Conventional TEM micrographs for the akaganeite before and after treating As^III^- and As^V^-spiked aqueous solutions (C_As_ ≈ 100 mg L^-1^, pH ≈ 3): Aka (black), Aka_As^III^_pH3_100 (wine red) and Aka_As^V^_pH3_100 (light red), respectively.


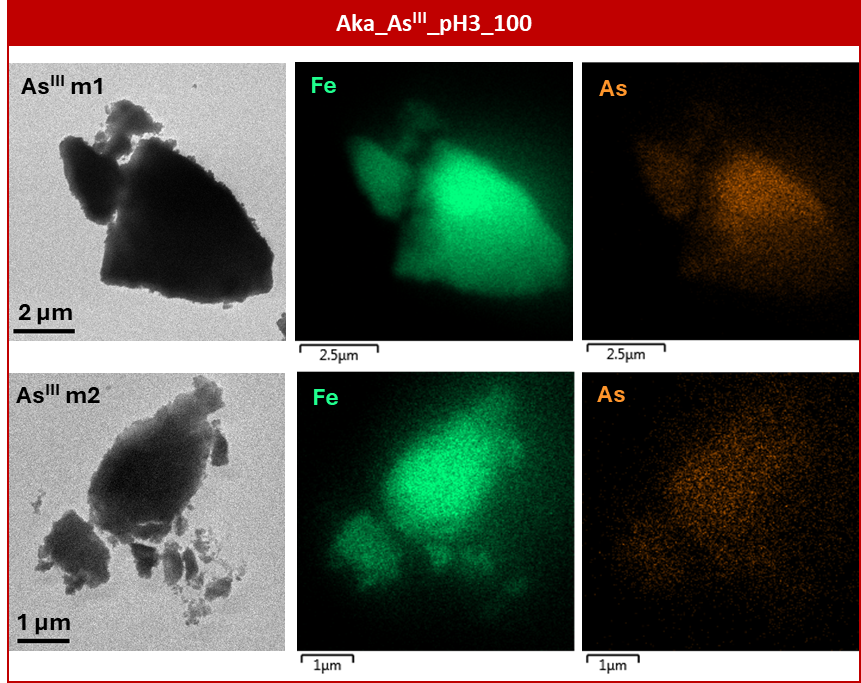


Figure S8 EDXS chemical mapping of the Aka_As^III^_pH3_100 sample at low magnification.


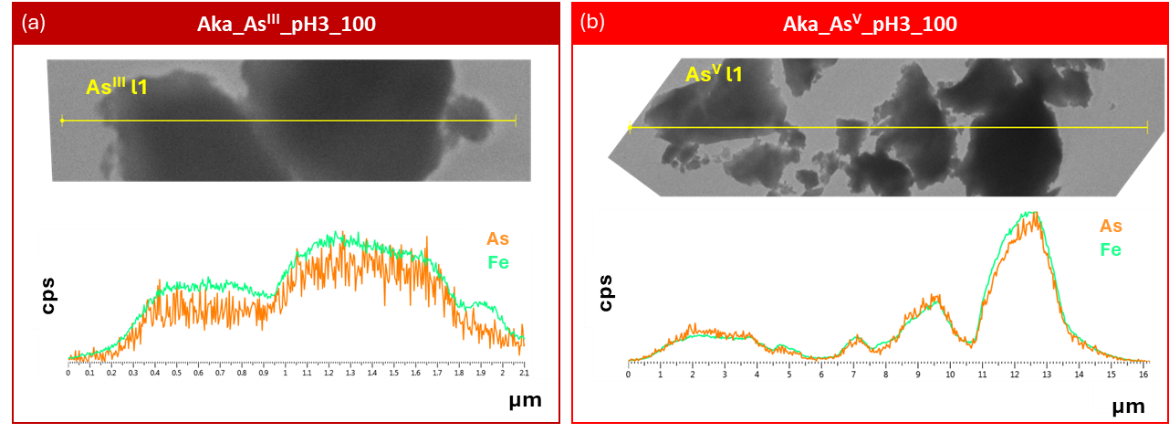


Figure S9 EDXS linear profile of the Aka_As^III^_pH3_100 (a) and Aka_As^V^_pH3_100 (b) samples at low magnification.


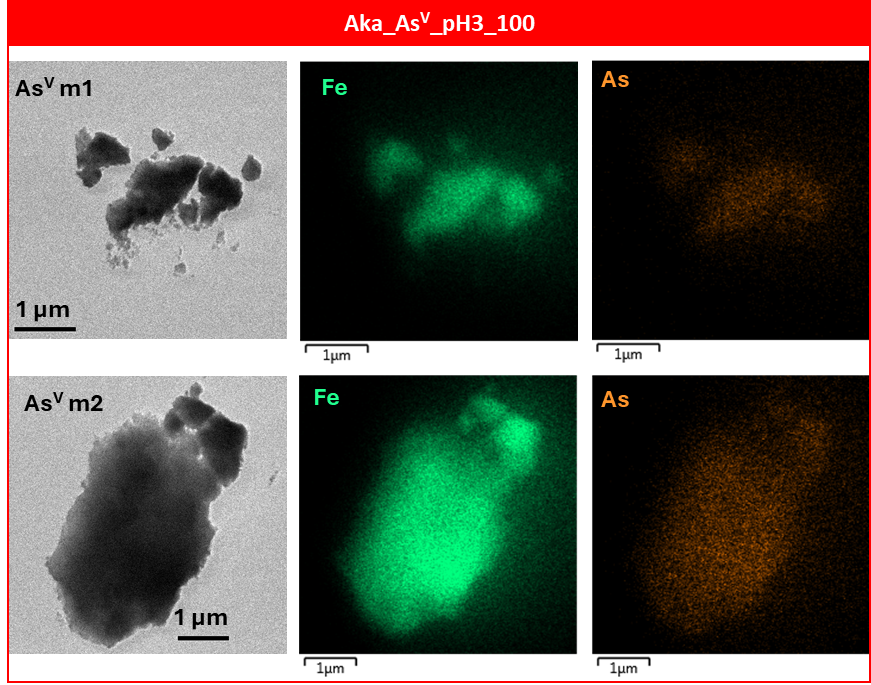


Figure S10 EDXS chemical mapping of the Aka_As^V^_pH3_100 sample at low magnification.

Table S4 EDXS data for the chemical mapping and linear profile of the Aka_As^III^_pH3_100 sample.

|  | **m1** | | | **m2** | | | **l1** | | |
| --- | --- | --- | --- | --- | --- | --- | --- | --- | --- |
| **Element** | Wt% | Wt% Sigma | Atomic % | Wt% | Wt% Sigma | Atomic % | Wt% | Wt% Sigma | Atomic % |
| **Fe** | 94.85 | 0.04 | 96.11 | 95.33 | 0.07 | 96.48 | 95.20 | 0.07 | 96.38 |
| **As** | 5.15 | 0.04 | 3.89 | 4.67 | 0.07 | 3.52 | 4.80 | 0.07 | 3.62 |
| **As/Fe (wt%) from EDXS** | 0.0543(4) | | | 0.0490(8) | | | 0.0504(8) | | |

Table S5 EDXS data for the chemical mapping and linear profile of the Aka_As^V^_pH3_100 sample.

|  | **m1** | | | **m2** | | | **l1** | | |
| --- | --- | --- | --- | --- | --- | --- | --- | --- | --- |
| **Element** | Wt% | Wt% Sigma | Atomic % | Wt% | Wt% Sigma | Atomic % | Wt% | Wt% Sigma | Atomic % |
| **Fe** | 92.09 | 0.10 | 93.98 | 91.82 | 0.06 | 93.78 | 92.57 | 0.05 | 94.35 |
| **As** | 7.91 | 0.10 | 6.02 | 8.18 | 0.06 | 6.22 | 7.43 | 0.05 | 5.65 |
| **As/Fe (wt%) from EDXS** | 0.086(1) | | | 0.0891(7) | | | 0.0803(6) | | |


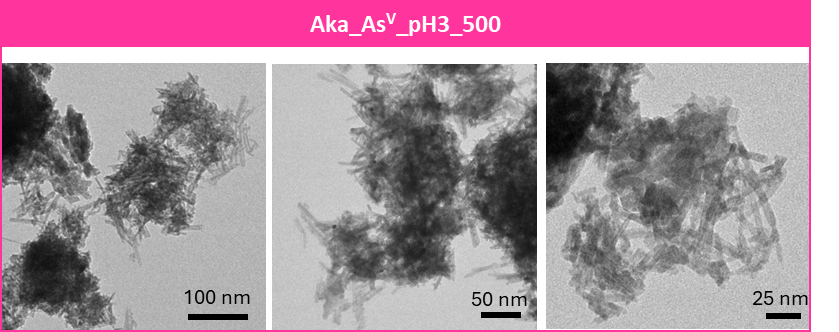


Figure S11 Conventional TEM micrographs for the akaganeite after treating As^V^-spiked aqueous solutions (C_As_ ≈ 500 mg L^-1^, pH ≈ 3).


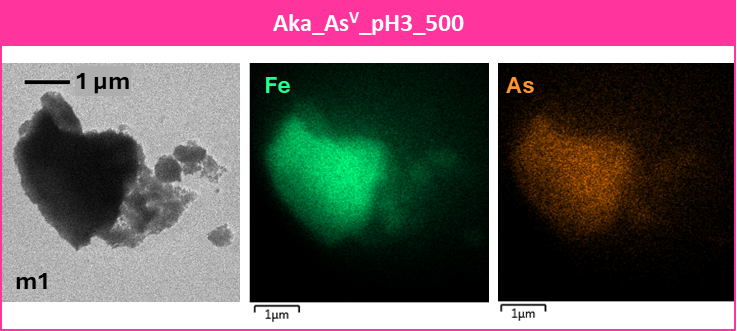


Figure S12 EDXS chemical mapping of the Aka_As^V^_pH3_500 sample at low magnification.


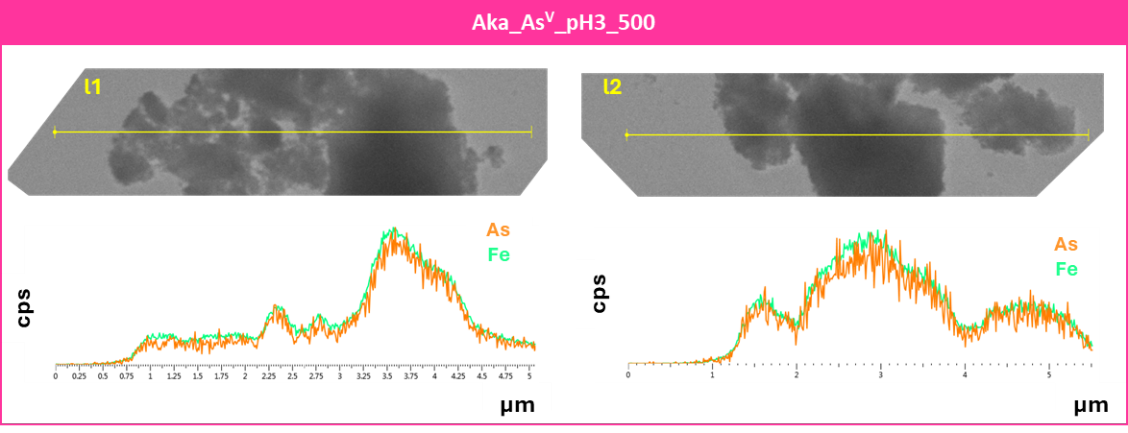


Figure S13 EDXS linear profiles of the Aka_As^V^_pH3_500 sample at low magnification.

Table S6 EDXS data for the chemical mapping and linear profile of the Aka_As^V^_pH3_500 sample.

|  | **m1** | | | **l1** | | | | **l2** | | |
| --- | --- | --- | --- | --- | --- | --- | --- | --- | --- | --- |
| **Element** | Wt% | Wt% | Atomic % | Wt% | Wt% | Atomic % | Wt% | | Wt% | Atomic % |
| **Fe** | 85.43 | 0.07 | 88.72 | 84.03 | 0.13 | 87.59 | 85.03 | | 0.17 | 88.40 |
| **As** | 14.57 | 0.07 | 11.28 | 15.97 | 0.13 | 12.41 | 14.97 | | 0.17 | 11.60 |
| **As/Fe (wt%) from EDXS** | 0.171(1) | | | 0.190(2) | | | 0.176(2) | | | |

# High-angle annular dark field detector (HAADF-STEM) in scanning TEM (STEM) mode


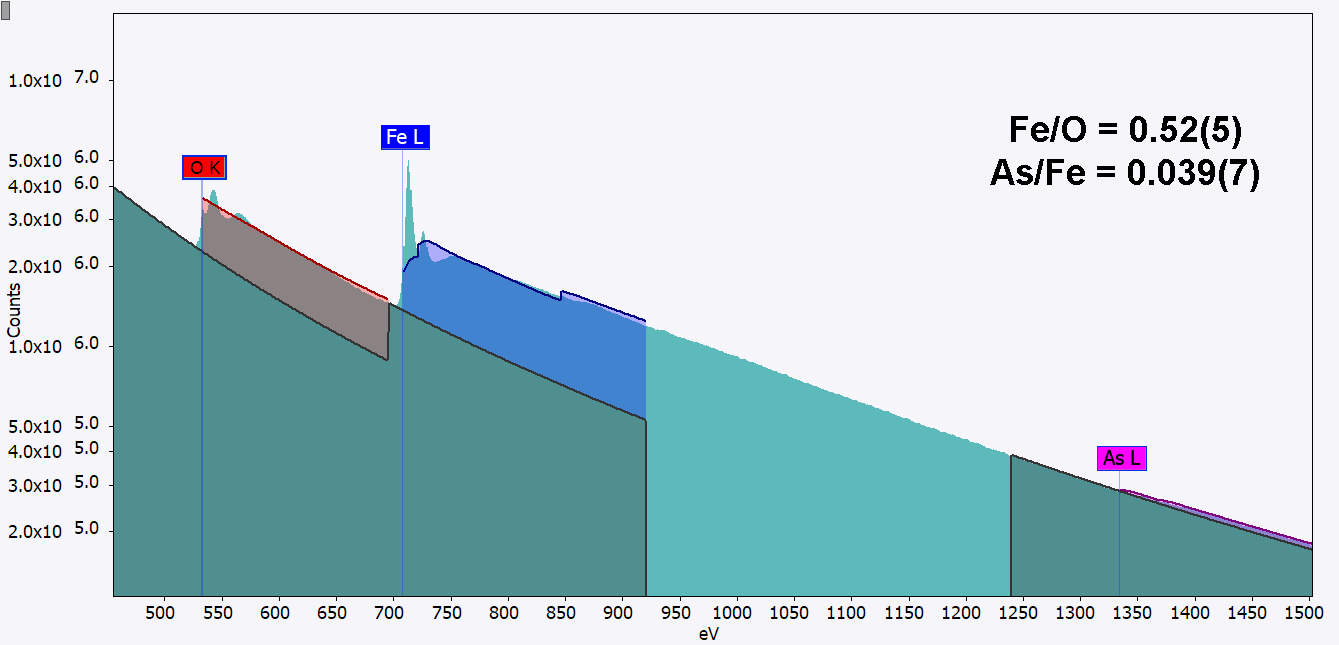


Figure S14 Average EEL spectrum from the Aka_AsV_pH3_150 sample (acquisition time: 100 s). The O-K, Fe-L and As-L edge are quantified with Hartree-Slater models and a power law background. Intensity is in log-scale to better show the As signal at ∼1330 eV. Quantification results were obtained by averaging 3 different regions in the sample.


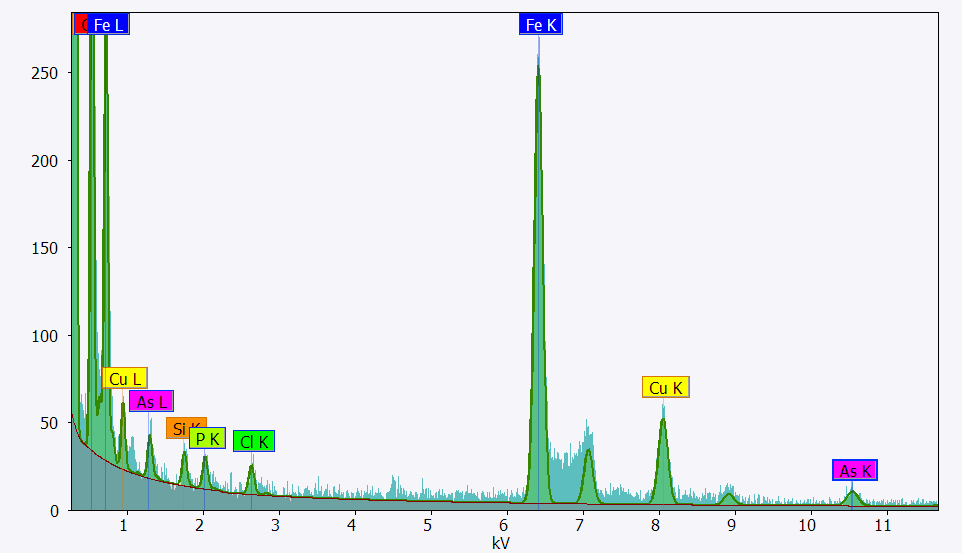


Figure S15 Average EDXS spectrum from one nanorod of the Aka_AsV_pH3_150 sample. The As/Fe ratio resulted 0.045(3) in good agreement with EELS data.

# DC magnetometry

**
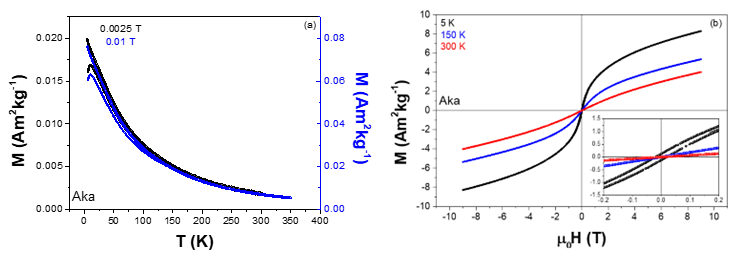
**

Figure S16 Zero field cooled – field cooled (ZFC-FC) curves recorded under 0.0025 and 0.01 T of external magnetic field (a) and magnetic isotherms at 5 K, 150 K, and 300 K (b) for the pristine akaganeite sample.

Figure S17 ZFC-FC curves of the pristine akaganeite and of the Aka_As^V^_pH3_100 and Aka_As^III^_pH3_100 samples.

Figure S18 ZFC-FC curves normalised (left) and absolute magnetisation values (right) for the Aka_As^V^_pH3_100 akaganeite sample recorded under different applied magnetic fields (0.01 T, 0.1 T).

Figure S19 ZFC-FC curves of the pristine akaganeite (Aka, black lines) and of the akaganeite samples recovered after treating As^III^- and As^V^-spiked aqueous solutions with different As concentrations.

Figure S20 ZFC curves (left) and ZFC-FC curves (right) of the pristine akaganeite (Aka, black lines) and of the akaganeite samples recovered before and after treating As^III^- and As^V^-spiked aqueous solutions with different As concentrations presented as separate curves.


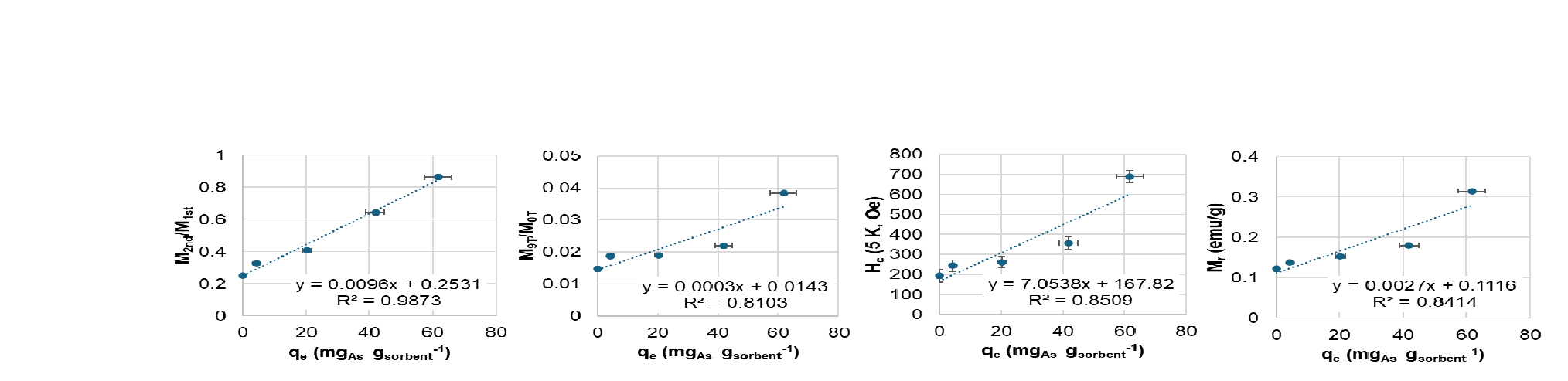


Figure S21 Linear dependence of different magnetic parameters from the amount of As adsorbed per unit mass of solid sorbent (q_e_).

# Characterization of the reference sample Aka_As^V^_6%


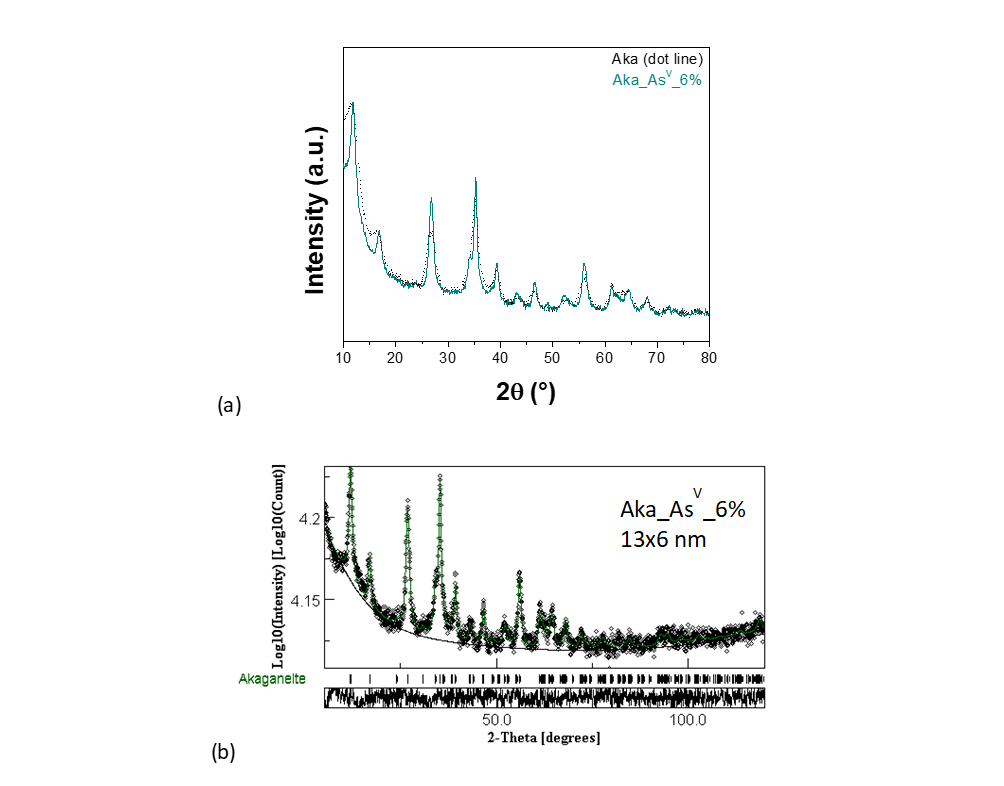


Figure S22 Powder XRD pattern (a) and Rietveld analysis (b) of the reference sample Aka_As^V^_6%.


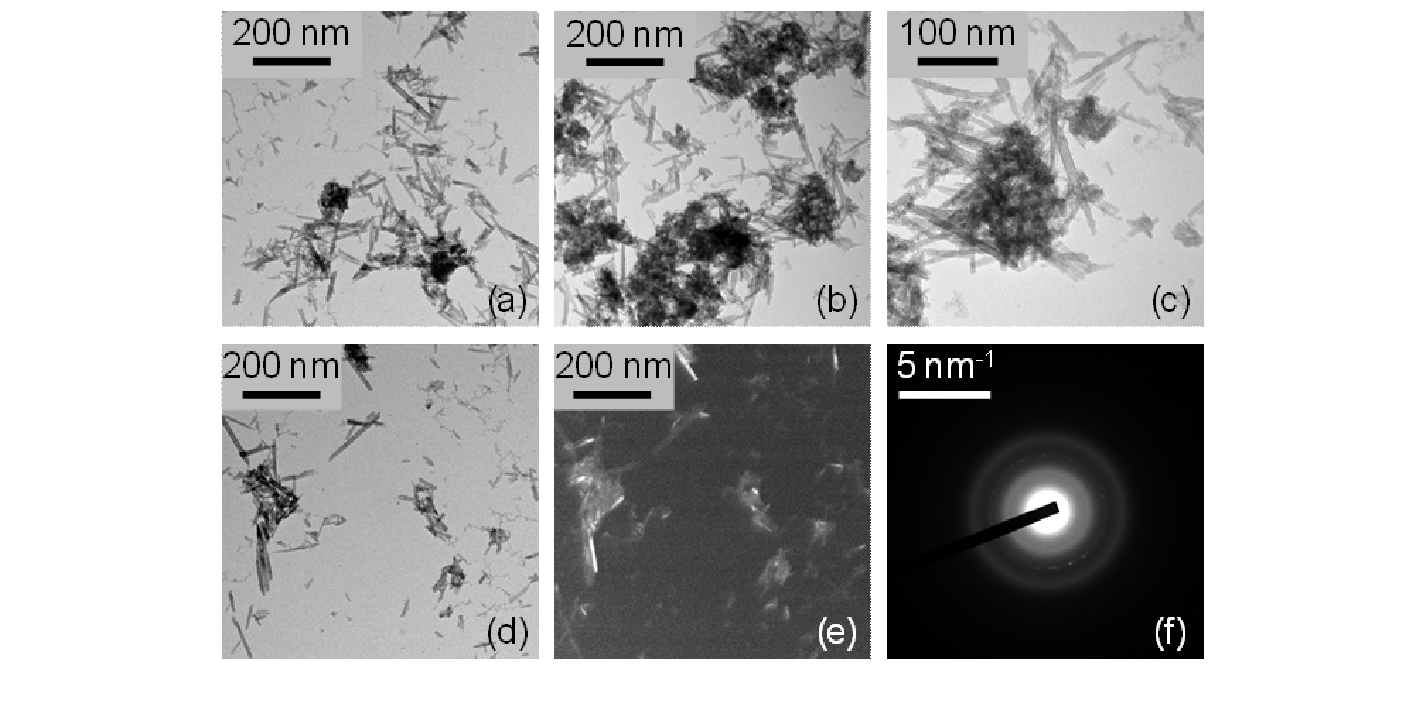


Figure S23 Bright field (a-d) and dark field (e) TEM micrographs and selected area electron diffraction (f) of the reference sample Aka_As^V^_6%.


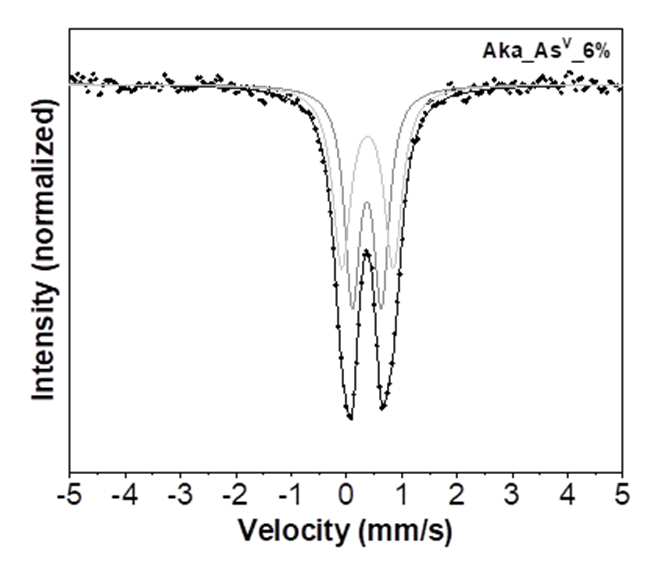


Figure S24 ^57^Fe Mössbauer spectrum of the reference sample Aka_As^V^_6%.

Figure S25 Zero field cooled – field cooled curves (a) and magnetic isotherms at 300 K (b) and 5 K (c) of the reference sample Aka_As^V^_6%.

# Magnetic characterization of the sample Aka_As^V^_pH3_250.

**
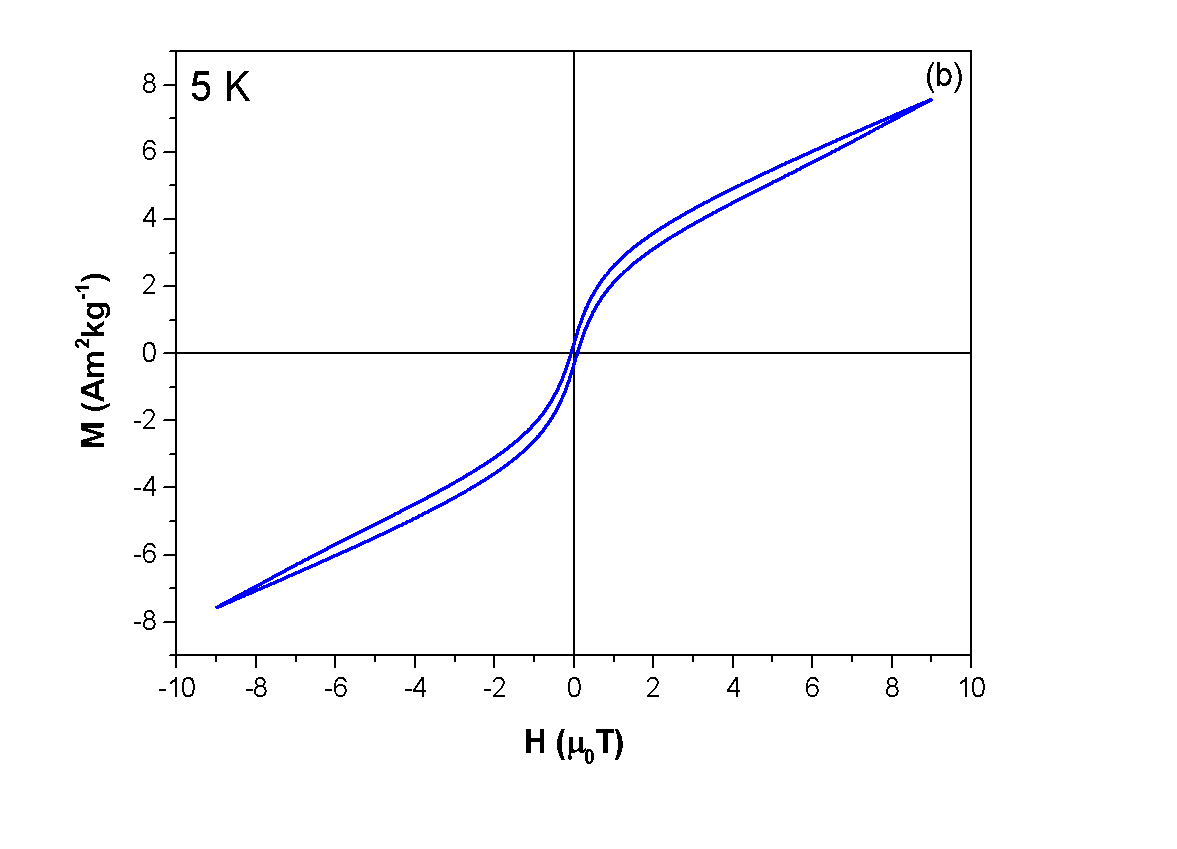
**

Figure S26 Magnetic characterization of the Aka_As^V^_pH3_250 sample.

# X-ray photoelectron spectroscopy (XPS)

Table S7 XPS data extracted from the XPS spectra of the akaganeite before and after the treatment of As^III^- and As^V^-spiked aqueous solutions (C_As_ ≈ 100, 150 mg L^-1^, pH ≈ 3). N.A. stands for “*not acquired*”.

|  | **E_b_ (eV)** | | | **Relative atomic concentration (%)** | | | | | **Fe/O** | **Cl/Fe** | **As/Fe** |
| --- | --- | --- | --- | --- | --- | --- | --- | --- | --- | --- | --- |
| **Sample** | **Fe 2p** | **As 3d** | **Fe 3p** | **C** | **As** | **Cl** | **Fe** | **O** |  |  |  |
| Aka | 711.4 | *N.A.* | *N.A.* | 21.5 | *N.A.* | 7.6 | 15.9 | 55.1 | 0.29 | 0.48 | *N.A.* |
| Aka_As^III^_pH3_100 | 711.4 | 45.7 | 56.9 | 21.0 | 2.1 | 4.1 | 14.3 | 58.4 | 0.25 | 0.29 | 0.18 |
| Aka_As^V^_pH3_150 | 712.6 | 45.8 | 57.7 | 25.6 | 2.4 | 2.7 | 15.5 | 53.7 | 0.29 | 0.17 | 0.22 |

Table S8 XPS data acquired by fitting the O 1s band of the XPS spectra of the akaganeite before and after the treatment of As^III^- and As^V^-spiked aqueous solutions (C_As_ ≈ 100, 150 mg L^-1^, pH ≈ 3).

| **Sample** | **Eb (eV)** | | | **Fe-OH_lattice_/Fe-O** |
| --- | --- | --- | --- | --- |
|  | **Fe-O** | **Fe-OH_lattice_** | **Fe-OH_ads_** |  |
| Aka | 529.6 | 531.0 | 532.1 |  |
| peak area % | 28.77 | 26.45 | 44.77 | **0.92** |
| Aka_As^III^_pH3_100 | 529.2 | 530.6 | 532.6 |  |
| peak area % | 36.89 | 44.46 | 18.66 | **1.21** |
| Aka_As^V^_pH3_150 | 529.4 | 530.4 | 532.2 |  |
| peak area % | 41.12 | 54.30 | 4.58 | **1.32** |
| **average Eb** | **529.4** | **530.7** | **532.3** |  |
| **standard deviation** | 0.2 | 0.2 | 0.2 |  |

# DC/AC magnetometry

To further investigate the nature of the magnetic signature observed for the As^V^-loaded akaganeite samples as second peak in the ZFC curve, an additional study was conducted by DC/AC magnetometry.

First, an aliquot of the pristine akaganeite sample was put in contact with an aqueous solution (milliQ water) at pH 3, whose pH was adjusted by dropping 0.01-0.1 M HCl solutions until the desired value was reached. The sample was named Aka_H_2_OmilliQ_pH3. The ZFC-FC curves for this sample (**Figure S27**) showed just a slight shift of the first peak in the ZFC curve with respect to that of the pristine akaganeite from about 11 K to 15 K, as also observed for the As-loaded samples. The observed behaviour might be interpreted as due to structural modification of the akageneite as response to a possible increase in the concentration of the chloride ions that are known to be hosted in the channels of the akageneite crystalline structure.

Figure S27 ZFC-FC curves of the sample Aka_H_2_OmilliQ_pH3 at a magnetic field of 0.01 T.

Secondly, the ZFC-FC curves were recorded also for the sample Aka_As^V^_pH8_100 (q_e_ = 42.3 mg_As_ g_sorbent_^-1^, **Figure S28**), which is analogous to the Aka_As^V^_pH3_100 but with the As removal test performed at pH 8. Two peaks are present in the ZFC curve with a quite similar trend except for the relative ratio between the magnetization values at the first and second peak, revealing that also in this case DC magnetometry can effectively probe the specific adsorbate-adsorbent interactions, through a magnetic response that is not only dependent on the adsorbate amount, but also on the speciation and on the environmental conditions, such as the pH.

Figure S28 ZFC-FC curves for the Aka_As^V^_pH8_100 sample in comparison with those of the Aka_As^V^_pH3_100 sample at two different magnetic field values (0.01 T, 0.1 T).

To understand which kind of magnetic system is at the basis of the observed phenomenon associated with the presence of the second peak in the ZFC curve, the following additional measurements on the sample Aka_As^V^_pH3_100 were performed:

1. ZFC-FC curves recorded under different magnetic field (0.01 T - 0.5 T, **Figure S29**,**Figure S30**);
2. in-phase (χ') and out-of-phase (χ'') AC susceptibility as a function of temperature (5-240 K) at different frequencies (1-999 Hz, **Figure S32**);
3. memory effect experiments (ZFC curves recorded after 0, 2, and 4 hours pause at 100 K during the cooling process, **Figure S32**);
4. thermoremanent magnetization (TRM) with 1-hour stops at 200 K and 100 K during the cooling process (**Figure S33**);
5. isothermal remanent magnetization (IRM) curves at different temperatures (5 K, 100 K, 300 K, **Figure S34**).

Figure S29 ZFC-FC curves recorded under different magnetic field (0.01 T - 0.5 T) on the sample Aka_As^V^_pH3_100 by a SQUID magnetometer (MPMS3XL from Quantum Design).

Figure S30 ZFC-FC curves recorded under different magnetic field (0.01 T - 0.5 T) on the sample Aka_As^V^_pH3_100 by a SQUID magnetometer (MPMS3XL from Quantum Design).

Figure S31 In-phase (χ') and out-of-phase (χ'') AC susceptibility as a function of temperature (5-240 K) at different frequencies (1-999 Hz) on the sample Aka_As^V^_pH3_100 recorded by a SQUID magnetometer (MPMS3XL from Quantum Design).

Figure S32 Memory effect experiments recorded on the sample Aka_AsV_pH3_100 by a VSM of a PPMS (PPMS9 from Quantum Design).

Figure S33 Thermoremanent magnetization (TRM) curve with 1-hour stops at 200 K and 100 K during the cooling process recorded on the sample Aka_AsV_pH3_100 by a VSM of a PPMS (PPMS9 from Quantum Design).

Figure S34 Isothermal remanent magnetization (IRM) curves at different temperatures (5 K, 100 K, 300 K) recorded on the sample Aka_AsV_pH3_100 by a SQUID magnetometer (MPMS3XL from Quantum Design).

The results showed: (i) the absence of a sharp cusp in FC but instead monotonic increase of FC magnetization with decreasing temperature (**Figure S29**, **Figure S30**); (ii) broad χ' AC susceptibility peak with slight changes with the frequency in the 100-200 K range (**Figure S32,** please, for further details refer to the paragraph entitled “*AC susceptibility analysis*”, **Table S9**); (iii) a weak memory effect (**Figure S32**); (iv) TRM curve without dips at the waiting temperatures (**Figure S33**); and (v) Mydosh parameter value (≈ 0.03) not compatible with a non-interacting superparamagnetic (SPM) or conventional (canonical) spin glass (SG) systems but rather with super spin glass (SSG) like or cluster-glass (CG) like states (please, for further details refer to the paragraph entitled “*AC susceptibility analysis*”, **Table S10**).

Thus, in the interpretation of these data we must take into account: (i) the structural nature of the system (akaganeite nanorods coated by an As^V^-bonded –(Fe-O-Fe)- surface framework); (ii) the possible occurrence of interparticle dipolar interactions between the As-bonded akaganeite nanorods; and (iii) the antiferromagnetic order of the akageneite with canted spins at the surface.

Based on the above premise, the system cannot be regarded as a “conventional” superparamagnets (SPM) or spin glasses (SG). Instead, the data are most consistent with a more complex interacting cluster glass (CG) like state, arising from the local disrupting of the main magnetic behaviour due to the presence of As-O-Fe bonds at the surface forming small magnetic islands.

In particular, given the nature of the investigated system, a simple superparamagnetic (SPM) state appears unlikely since superparamagnetism presupposes ferro- or ferrimagnetic single-domain particles, whereas akaganeite itself is susually described as an antiferromagnet with canted spins. Both interparticle dipolar interactions and surface spin disorder are expected to contribute significantly to the overall magnetic behavior due to the possible contact between the particles. Therefore, more complex collective magnetic states must be considered.

The analysis of the frequency-dependent AC susceptibility measurements was carried out by standard models, including Arrhenius and Vogel–Fulcher laws, together with an evaluation of the Mydosh parameter. While the Arrhenius model is not strictly appropriate for interacting nanoparticle systems, it provides a useful reference. As expected, both Arrhenius and Vogel–Fulcher fits yielded rather unphysical parameters, which is a common artefact when applied to the systems with complex collective dynamics. In particular, when constraining the attempt time to physically reasonable values (~10⁻⁹ s), the resulting activation barriers reached values on the order of thousands of Kelvin, which are unrealistic for this type of system. The Mydosh parameter (relative shift of the maximum per frequency decade) was estimated to be δTf ≈ 0.03, significantly larger than values typical of canonical spin glasses and instead lying in the range commonly associated with cluster-glass or strongly interacting SPM systems. Consequently, AC susceptibility alone does not allow an unambiguous distinction between SSG-like or CG-like states.

In the TRM protocol, the field-cooled magnetization was measured under an applied field of 10 mT down to 5 K, with intermittent 1-hour stops at selected temperatures. During each stop, the system was allowed to relax for the waiting time (aging), and upon resuming cooling, rejuvenation occurs. When the external magnetic field is switched off, magnetization decay is expected both in non-interacting SPM systems and in weakly or strongly interacting nanoparticle assemblies.^[15]^ Upon reheating, the temperature dependence of magnetization may reveal memory of the previous cooling process, visible as steps corresponding to relaxation at the waiting temperatures, as demonstrated by Sun et alii.^[16]^ However, this type of memory effect is qualitatively similar for both SPM and SSG systems, as first discussed by Sasaki et al.^[15]^ and later observed in related systems, including our previous work.^[17]^ Therefore, TRM memory alone does not uniquely identify the interaction regime. In our measurements, no pronounced dips at the waiting temperatures were observed, suggesting the absence of strong collective freezing.

Thus, memory effect in the ZFC protocol was investigated. A standard ZFC curve was first measured in an external field of 10 mT. The sample was then cooled again with an intermediate stop at a selected temperature, and a second ZFC curve was recorded. The difference between the two curves was subsequently evaluated. For an ideal non-interacting ensemble of particles, the ZFC magnetization is independent of the waiting time. In contrast, SSG systems exhibit waiting-time-dependent relaxation, manifested by a cusp in the subtracted curve at the waiting temperature.^[15]^ It has been shown that real SPM systems may display weak waiting-time dependence in TRM but only minor effects in ZFC, whereas SSG systems show strong dependence in both protocols.^[15]^ In SSG-like systems, the ZFC magnetization becomes effectively independent of waiting time on experimental time scales.^[17,18]^ Thus, a pronounced memory effect in the genuine ZFC protocol is considered characteristic primarily of true SSG behavior. In our data, the ZFC memory effect is weak, and this favours an SSG-like rather than a true SSG state.

Nevertheless, we have to point out that the SSG state requires the formation of a single-domain regime with interactions between these domains (typically single-domain particles), which is not very probable for akaganeite, as it exhibits AFM ordering at much lower temperatures.

In the alternative CG scenario, groups of spins form locally ordered magnetic clusters that interact collectively, analogous to superspins in SSG systems. A commonly reported feature of CG systems is a monotonic increase of FC magnetization with decreasing temperature.^[19]^

Taking into account the structural characteristics of the sample, the glassy magnetic phase is located predominantly at the nanoparticle surface (As-diluted layer), while the interparticle distance remains sufficiently small to enable dipolar interactions, the observed magnetic response represents a superposition of an intrinsic surface spin-disordered (glassy-like) state modulated by time-dependent dipolar fields arising from the interacting nanoparticle assembly, and might be compatible with a CG state.

Nevertheless, another hypothesis might be the occurrence of a Griffiths-phase-like precursor clustering within the disordered surface layer.

# AC susceptibility analysis

Table S9 Temperature of the maxima of the in-phase (χ') AC susceptibility for the given attempt frequency.

| **Frequency (Hz)** | **Temperature (K)** |
| --- | --- |
| 1 | 164.98 |
| 5.62 | 169.99 |
| 31.62 | 175.00 |
| 177.88 | 179.99 |
| 999.04 | 180.00 |


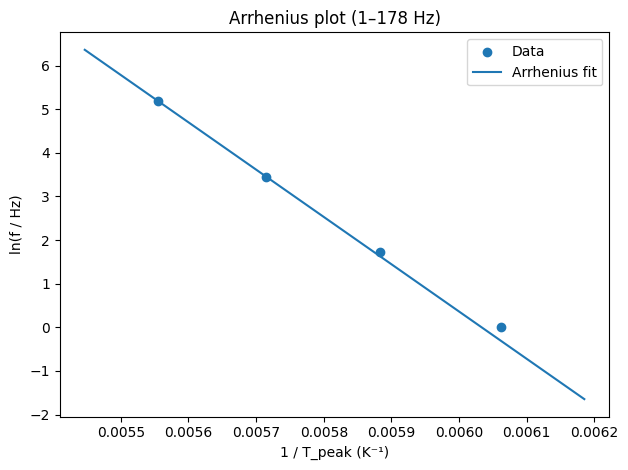

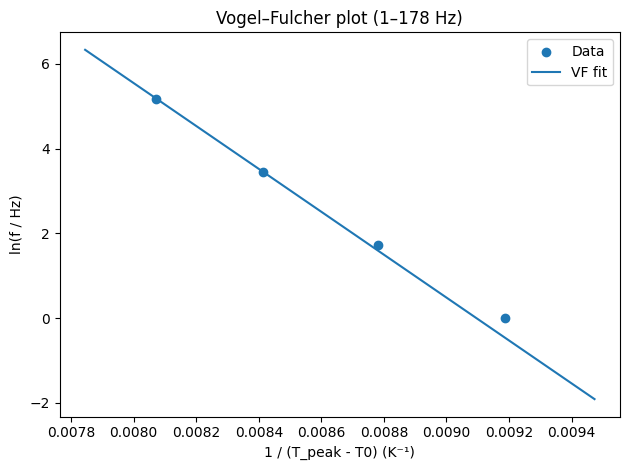


Figure S35 Fit of the data extracted from the maxima of the in-phase (χ') AC susceptibility by the Arrhenius and Vogel-Fulcher laws.


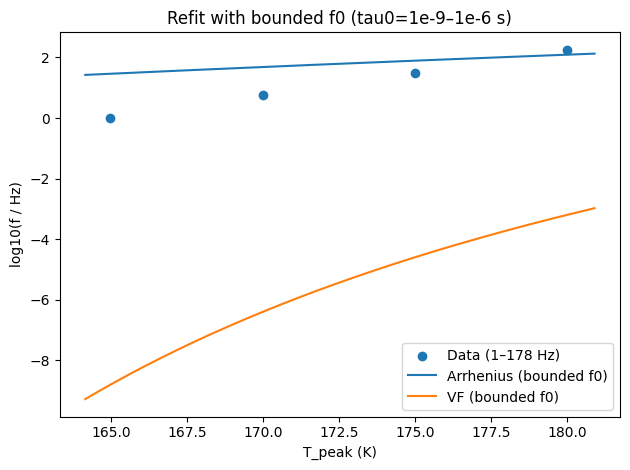


Figure S36 Fit of the data extracted from the maxima of the in-phase (χ') AC susceptibility by the Arrhenius and Vogel-Fulcher laws with the limits for the expected range of relaxation times (upper bound for τ = 10^-9^ s).

Table S10 Equations used for the AC susceptibility analysis.

| **Name** | **Equation** | **Parameters** |
| --- | --- | --- |
| Arrhenius law (Néel- Arrhenius) | $\tau= \tau_{0}exp\left( \frac{E_{a}}{k_{B}T} \right)\Longleftrightarrow f= \frac{1}{2\pi\tau_{0}}exp\left( -\frac{E_{a}}{k_{B}T} \right)$ | $\tau$ is the relaxation time  $\tau_{0}$ is the microscopic attempt time  $f$ is the measurement frequency  $E_{a}$ is the energy barrier  $k_{B}$ is the Boltzmann constant  $T$ is the temperature |
| Vogel-Fulcher law | $\tau= \tau_{0}exp\left( \frac{E_{a}}{k_{B}(T-T_{0})} \right)\Longleftrightarrow f= \frac{1}{2\pi\tau_{0}}exp\left( -\frac{E_{a}}{k_{B}(T-T_{0}} \right)$ | $\tau$ is the relaxation time  $\tau_{0}$ is the microscopic attempt time  $f$ is the measurement frequency  $E_{a}$ is the energy barrier  $k_{B}$ is the Boltzmann constant  $T$ is the temperature  $T_{0}$ is the Vogel-Fulcher temperature (interaction/freezing temperature) |
| Mydosh parameter | $\delta T_{f}=\frac{\Delta T_{f}}{T_{f}\Delta logf}$ | $\delta T_{f}$ is the Mydosh parameter (relative frequency shift)  $T_{f}$ is the freezing/blocking temperature (χ' peak)  $\Delta T_{f}$ is the change of $T_{f}$ between frequencies  $\Delta logf$ is the change in logarithm of frequency  $f$ is the measurement frequency |

# References

[1] E. . Deliyanni, D. . Bakoyannakis, A. . Zouboulis, K. . Matis, *Chemosphere* **2003**, *50*, 155.

[2] M. Thommes, K. Kaneko, A. V Neimark, J. P. Olivier, F. Rodriguez-Reinoso, J. Rouquerol, K. S. W. Sing, *Pure Appl. Chem.* **2015**, *87*, 1051.

[3] K. S. Lin, Z. P. Wang, S. Chowdhury, A. K. Adhikari, *Thin Solid Films* **2009**, *517*, 5192.

[4] H. Simon, G. Cibin, I. Freestone, E. Schofield, *J. Phys. Condens. Matter* **2021**, *33*, DOI 10.1088/1361-648X/ac08b6.

[5] N. Chubar, M. Szlachta, V. Gerda, *J. Environ. Chem. Eng.* **2025**, *13*, DOI 10.1016/j.jece.2025.115748.

[6] G. Morin, G. Ona-Nguema, Y. Wang, N. Menguy, F. Juillot, O. Proux, F. Guyot, G. Calas, G. E. Brown, *Environ. Sci. Technol.* **2008**, *42*, 2361.

[7] V. Mameli, A. Musinu, D. Niznansky, D. Peddis, G. Ennas, A. Ardu, C. Lugliè, C. Cannas, *J. Phys. Chem. C* **2016**, *120*, 27635.

[8] C. Cara, V. Mameli, E. Rombi, N. Pinna, M. Sanna Angotzi, D. Nižňanský, A. Musinu, C. Cannas, *Microporous Mesoporous Mater.* **2020**, *298*, 110062.

[9] M. Sanna Angotzi, V. Mameli, A. Fantasia, C. Cara, F. Secci, S. Enzo, M. Gerina, C. Cannas, *Nanomaterials* **2022**, *12*, 326.

[10] R.M.Cornell ; U. Schwertmann, R. M. Cornell, U. Schwertmann, *The Iron Oxides*, Wiley-VCH Verlag GmbH & Co. KGaA, Weinheim, FRG, **1996**.

[11] E. Murad, *Clay Miner.* **1979**, *14*, 273.

[12] D. Chambaere, E. De Grave, *J. Magn. Magn. Mater.* **1984**, *42*, 263.

[13] D. Rezel, J. M. R. Genin, *Hyperfine Interact.* **1990**, *57*, 2067.

[14] V. Villacorta, C. A. Barrero, M.-B. Turrión, F. Lafuente, J.-M. Greneche, K. E. García, *RSC Adv.* **2020**, *10*, 42688.

[15] M. Sasaki, P. E. Jönsson, H. Takayama, H. Mamiya, *Phys. Rev. B* **2005**, *71*, 104405.

[16] Y. Sun, M. B. Salamon, K. Garnier, R. S. Averback, *Phys. Rev. Lett.* **2003**, *91*, 167206.

[17] B. Bittova, J. Poltierova Vejpravova, M. P. Morales, A. G. Roca, A. Mantlikova, *J. Magn. Magn. Mater.* **2012**, *324*, 1182.

[18] P. S. A. Kumar, P. A. Joy, S. K. Date, *J. Phys. Condens. Matter* **1998**, *10*, L487.

[19] H. Szymczak, M. Baran, G.-J. Babonas, R. Diduszko, J. Fink-Finowicki, R. Szymczak, *J. Magn. Magn. Mater.* **2005**, *285*, 386.
